# Metallic Copper-Based Dual-Enzyme Biomimetic Nanoplatform for Mild Photothermal Enhancement of Anticancer Catalytic Activity

**DOI:** 10.34133/bmr.0034

**Published:** 2024-06-05

**Authors:** Ziqun Chen, Ying Li, Qi Xiang, Yunfang Wu, Haitao Ran, Yang Cao

**Affiliations:** Chongqing Key Laboratory of Ultrasound Molecular Imaging, Ultrasound Department of the Second Affiliated Hospital of Chongqing Medical University, Institute of Ultrasound Imaging, State Key Laboratory of Ultrasound in Medicine and Engineering of Chongqing Medical University, Chongqing 400016, China.

## Abstract

**Background:** Chemodynamic therapy (CDT) is recognized as a promising cancer treatment. Recently, copper sulfide nanostructures have been extensively employed as Fenton-like reagents that catalyze the formation of acutely toxic hydroxyl radicals (·OH) from hydrogen peroxide (H_2_O_2_). However, CDT therapeutic potency is restricted by the tumor microenvironment (TME), such as insufficient amounts of hydrogen peroxide, excessive glutathione levels, etc. To address these disadvantages, glucose oxidase (GOx) or catalase (CAT) can be utilized to enhance CDT, while low therapeutic efficacy still inhibits their future applications. Our previous study revealed that mild photothermal effect could boost the CDT catalytic effectiveness as well as GOx enzyme activity over a range.

**Results:** We engineered and constructed a hollow CuS nanoplatform loaded with GOx and CAT, coating with macrophage membranes (M@GOx-CAT@CuS NPs). The nanoplatforms allowed enhancement of the reactive oxygen species creation rate and GOx catalytic activeness of CDT through mild phototherapy directed by photoacoustic imaging. After actively targeting vascular cell adhesion molecule-1 (VCAM-1) in cancer cells mediated by macrophage membrane coating, M@GOx-CAT@CuS NPs released GOx and CAT under near-infrared irradiation. GOx catalyzed the formation of H_2_O_2_ and gluconic acid with glucose, creating a better catalytic environment for CDT. Meanwhile, CAT-catalyzed H_2_O_2_ decomposition to generate sufficient oxygen, appropriately alleviating the oxygen shortage in the TME. In addition, starvation effects decreased adenosine triphosphate levels and further underregulated heat shock protein expression to reduce the heat resistance of tumor cells, resulting in a better mild phototherapy outcome. Both in vitro and in vivo experiments demonstrated that the newly developed M@GOx-CAT@CuS nanoplatform has remarkable synergistic anticancer therapeutic effects.

**Conclusion:** The cascade reaction-enhanced biomimetic nanoplatform opens up a new avenue for precision tumor diagnostic and therapeutic research.

## Introduction

Cancers are among the most serious diseases that threaten human health [1,2]. Owing to the great complexity and heterogeneity of tumor microenvironment (TME), traditional monotherapy strategies (i.e., chemotherapy, surgery, radiotherapy) face challenges like low specificity, high recurrence rate, and unavoidable damage to normal tissues [3,4]. Recently, with the development of new strategies and therapeutic anticancer drugs, the trend of combination therapy for tumors is increasing year by year [5]. Photothermal therapy (PTT) aims to kill tumor cells by converting light energy into heat energy under the effect of near-infrared (NIR) light [6,7]. Numerous NIR-responsive photothermal materials have been researched in recent decades, including noble metal nanostructures, carbon-based nanomaterials, rare earth compounds, many organic nanoparticles and polymers, and semiconductor nanomaterials [8–10]. Among numerous photothermal conversion agents, hollow mesoporous copper sulfide nanoparticles (CuS NPs) have high photothermal conversion efficiency in the NIR 2-region bio-window, are biocompatible and degradable, are simple to synthesize, and the hollow mesoporous structure has a unique advantage in drug loading [11]. In addition, CuS NPs have chemodynamic properties that can promote the catalytic conversion of H_2_O_2_ to the more cytotoxic ·OH via Fenton or Fenton-like reactions [12,13].

Chemodynamic therapy (CDT) is recognized as a potential cancer approach and is rapidly evolving [14]. Under the mildly acidic TME, metal ions (e.g., Fe, Cu, Mo, and Mn) promote the catalytic conversion of intracellular hydrogen peroxide (H_2_O_2_) to extremely poisonous hydroxyl radicals (·OH) through Fenton or Fenton-like reactions to kill cancer cells [15–18]. Nevertheless, limited hydrogen peroxide levels and glutathione overexpression within tumor tissues make CDT catalytically less efficient, limiting its further application [19].

Therefore, much efforts are made to overcome their shortages. Glucose oxidase (GOx), as a natural enzyme, catalyzes the decomposition of glucose to generate H_2_O_2_ and gluconic acid, the products of which make up for a lack of raw materials for CDT and promote the occurrence of CDT [20,21]. More importantly, GOx disrupts glucose metabolism at the tumor site, thereby inhibiting tumor growth [22]. Since the efficiency of GOx catalysis relies on the oxygen level and the catalyzing temperature (43 to 60 °C), appropriately elevating the temperature at the tumor site is beneficial to increase the enzymatic activity of GOx as well as to increase the rate of the Fenton or Fenton-like reactions and to strengthen the combination of starvation therapy and CDT [23–25]. On the other hand, catalase (CAT) is an antioxidant enzyme that catalyzes H_2_O_2_ to O_2_ [26]. The released O_2_ not only promotes GOx to consume more glucose but also relieves tumor hypoxia [27].

It has reported the PTT and CDT combined treatment of tumors utilizing the photothermal effect to increase the catalytic productivity of CDT [28,29]. However, excessive temperatures (>50 °C) in this process were inevitably damage healthy tissue surrounding the tumor [30,31]. Therefore, as an alternative, mild PTT (mPTT) (<45 °C) keeps the temperature at a relatively low level and is more suitable for clinical applications [32,33]. However, heat stress-induced protective heat shock proteins (HSPs) in cancer cells can fix thermal damage and prevent tumor cells from apoptosis via multiple stress-related pathways, enhancing the heat resistance of tumors, which leads to the poor anticancer effect of mPTT [34]. It is well known that HSP expression is highly positively correlated with adenosine triphosphate (ATP) content [35]. By blocking the energy source, the GOx can effectively inhibit the production of intracellular ATP [36]. Motivated by the benefits of mPTT and starvation therapy combined therapy, new nanotherapeutic agents with mild photothermal effects, starvation therapy effects, and Fenton catalyst activity should be developed to accomplish remarkable antitumor therapy.

**Fig. 1.**
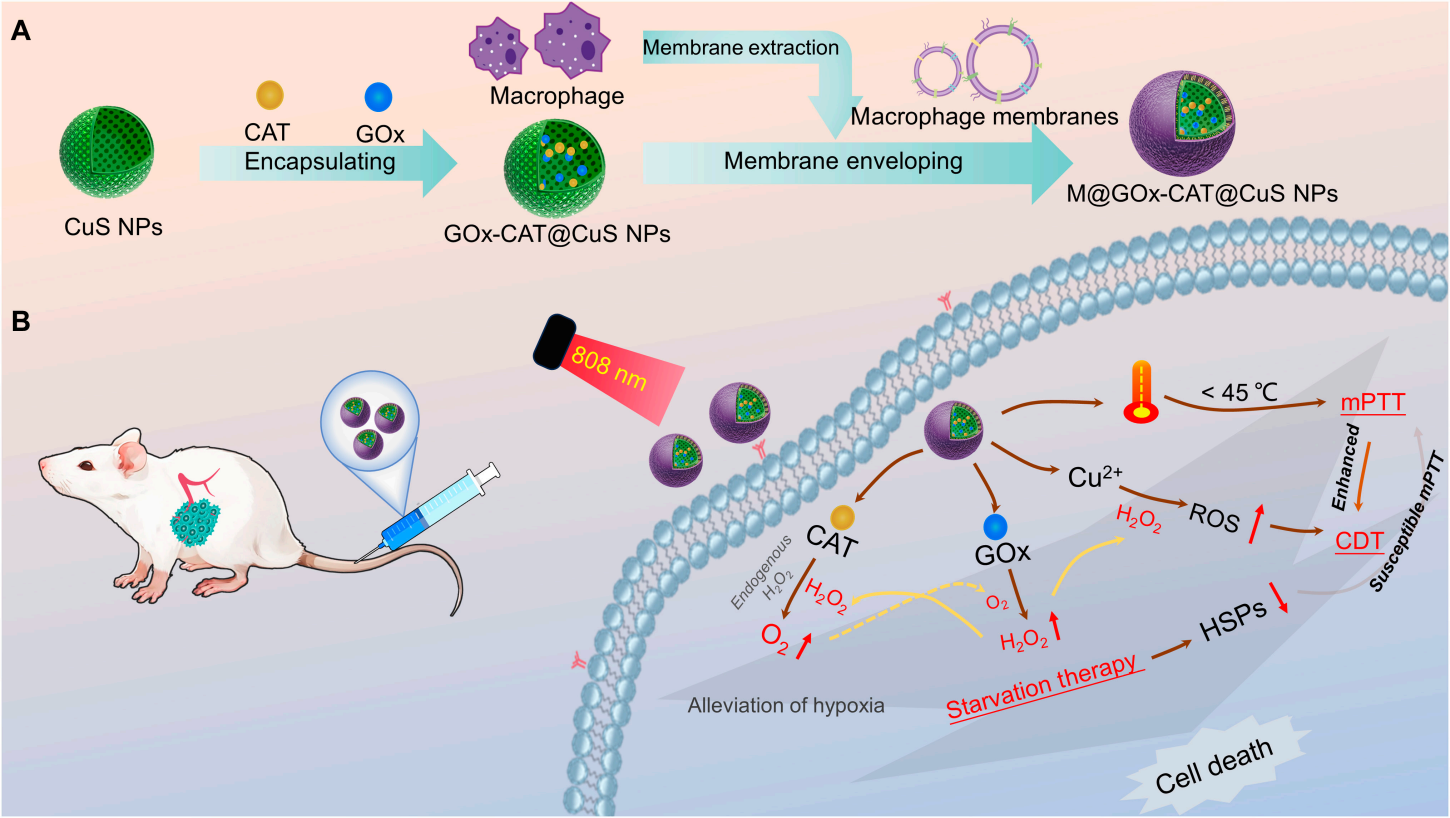
Schematic diagram of the (A) synthesis of M@GOx-CAT@CuS and (B) its enhancement of the efficiency of combined CDT and GOx/CAT treatment by mPTT.

To implement the above strategy, in this study, we constructed an intelligent NIR-responsive macrophage membrane-camouflaged cascade CuS-based nanosystems for mild photothermal enhancement of the efficacy of the combined CDT and GOx/CAT in the treatment of breast cancer (Fig. 1). Macrophage membranes can express integrin α4β1 interacting with vascular cell adhesion molecule-1 (VCAM-1) of breast cancer cells to promote nanoparticles internalization into the cells, leading to active targeted delivery of tumor tissue. Under the effect of NIR light, the nanoparticles released GOx and CAT within tumor sites. GOx was supposed to starve the tumor by consuming glucose. In addition, the hydrogen peroxide catalytically produced by GOx and the endogenous hydrogen peroxide decomposed in situ by CAT to generate adequate oxygen can further improve the effectiveness of starvation treatment and appropriately alleviate the lack of oxygen in TME. As GOx catalyzed the production of gluconic acid from glucose in TME, it further lowered the pH value of TME, which was conducive to the promotion of CDT. Additionally, starvation therapy decreased ATP levels and further down-regulated HSP expression. Reduced HSP expression reduced the heat tolerance of tumor cells, leading to improved mPTT treatment outcomes. The oxygen self-supply cascade response enhancement systems could fulfill mPTT-enhanced starvation and CDT therapy and compensate for each other to maximize precision tumor therapy.

## Materials and Methods

### Materials and reagents

Copper (II) chloride (CuCl_2_), polyvinylpyrrolidone (PVP-K40), GOx, and CAT were supplied by Aladdin Reagent Co., Ltd (Shanghai, China). Hydrazine hydrate (N_2_H_4_·H_2_O) and hydrogen peroxide (H_2_O_2_, 30%) were prepared by Chongqing Medical University. Membrane Protein Extraction Kit, 2',7'-dichlorodihydrofluorescein diacetate (DCFH-DA), and ATP detection reagent were obtained from Beyotime Biotechnology (Shanghai, China).

### Preparation of CuS NPs

The preparation of CuS NPs was based on former reports [37,38]. Briefly, in 50 ml of deionized (DI) water, 200 μl of CuCl_2_ (0.5 M) was diluted and 0.48 g of PVP-K40 was introduced. After agitating for 5 min at 25 °C, 50 ml of NaOH (0.01 mM, pH = 9) and 12.8 μl of N_2_H_4_·H_2_O were pipetted into the mixture. Cu_2_O nanospheres were obtained after blending for 5 min. After that, 400 μl of Na_2_S (320 mg/ml) was admixed, and the solution was stirred over 2 h at 65 °C. Centrifugation (11,000 rpm) for 10 min was performed to collect the CuS NPs, which were rinsed 3 times with DI water distributed in water.

### Extraction of the macrophage membranes

Raw264.7 cell membranes were extracted according to the Membrane Protein Extraction Kit. In short, 2 × 10^7^ cells were suspended in 1 ml of Membrane Protein Extraction Reagent A comprising 1 mM phenylmethylsulfonyl fluoride and placed in a freezing bath for 10 to 15 min. The cells were frozen and thawed 3 times in liquid nitrogen to disrupt the cells at room temperature, and then the cell disruption solution was centrifuged at 2,000 rpm at 4 °C for 10 min. The supernatant was collected and recentrifuged at 13,000 rpm for 30 min at 4 °C, and the precipitate was collected and stored at −80 °C. All these procedures were performed in a freezing bath.

### Preparation of M@GOx-CAT@CuS NPs

CuS (15 mg) was dispersed in a total of 5 mg of GOx and CAT solutions and placed in a magnetic stirrer for 24 h. The supernatant was purified by triple centrifugation (11,000 rpm, 10 min) to obtain GOx-CAT@CuS NPs. After mixing with macrophage membranes at a mass ratio of 4:1, the blend was sonicated for 10 min under a cold bath to complete the membrane encapsulation [39,40]. The M@GOx-CAT@CuS NPs were subsequently collected after centrifugation (11,000 rpm, 10 min) at 4 °C to eliminate redundant membranes.

### In vitro enzyme release

CuS NPs (20 mg) and 10 mg each of GOx and CAT were distributed in 10 ml of DI water under stirring for 24 h. The samples were then centrifuged at 11,000 rpm for 10 min and rinsed with DI water thrice. The supernatant was collected for measuring GOx and CAT loading efficiency. Then, the NPs were capped. The M@GOx-CAT@CuS NPs treated above were distributed in 7 ml of phosphate-buffered saline (PBS) solution and divided into 4 groups: pH = 7.4, pH = 6.5, pH = 7.4 + NIR, and pH = 6.5 + NIR. The experimental solutions of all groups were then put into a thermostatic shaker (37 °C, 100 rpm), and the groups requiring laser irradiation were given 808-nm laser treatment (1 W/cm^2^, 10 min) [41]. One milliliter of sample was removed at 0, 2, 4, 8, 12, 24, 48, and 72 h. After centrifugation, the concentration of the enzyme in the supernatant was estimated with the BCA Protein Concentration Assay Kit. After that, the amount of enzyme released at different time points can be further calculated.

The cumulative drug release rate is calculated as follows:

$$E_{r}=\frac{V_{e}\sum_{1}^{n-1}C_{i}+V_{0}C_{n}}{m_{\text{drug}}}\times 100\%$$

where *E_r_* is cumulative drug release; *V_e_* is the volumetric displacement of PBS; *V*_0_ is the total amount of fluid released; *C_i_* is the concentration of the liquid released at the instant of *i*; *m_drug_* is the total mass of drug within nanoparticles; and *n* is the number of times PBS was replaced.

### Evaluation of encapsulation and loading efficiency

The enzyme concentration in M@GOx-CAT@CuS NPs can be calculated by bicinchoninic acid (BCA) assay and thermogravimetric analysis (TGA) thermogravimetric analysis. The encapsulation efficiency (EE) and load efficiency (LE) of GOx and CAT in M@GOx-CAT@CuS NPs were determined as follows.

$$EE=\frac{m-m_{1}}{m}\times 100\%$$

$$LE=\frac{m-m_{1}}{m_{p}}\times 100\%$$

where *m*, *m*_1_, and *m_p_* are the total mass of added enzyme, the quality of enzyme in the solution supernatant, and the quality of nanoparticles, respectively.

### Evaluation of enzyme stability

To assess the stability of the enzymes at elevated temperatures, GOx and CAT were incubated in an aqueous bath for 15 min at 25, 37, and 45 °C, respectively. The enzyme activities were assayed through the GOx test kit and the CAT test kit, respectively. The M@GOx-CAT@CuS NPs suspensions, free CAT, and GOx solutions were placed at room temperature. The enzymatic activity was investigated at different time intervals with CAT and GOx assay kits.

### In vitro photothermal performance of M@GOx-CAT@CuS NPs

Put M@GOx-CAT@CuS NPs (400, 200, 100, 50, and 0 μg/ml), CuS NPs (200 μg/ml), and PBS into a 96-well plate and exposed to an 808-nm laser (0.75, 1, 1.25, 1.5, and 2 W/cm^2^, 10 min). The plate was imaged using an infrared thermal camera and monitored for temperature in real time. M@GOx-CAT@CuS NPs (200 μg/ml) were exposed to 5 repeated heating and cooling cycles under laser at 808 nm to explore the photothermal stability. We calculated the photothermal conversion efficiency (η) by the following formula.

$$\eta =\frac{hS\left(T_{\max}-T_{\min}\right)-Q_{s}}{I\left(1-10^{-A_{\lambda }}\right)}$$

where *h* is heat transmission coefficient; *S* is surface area of container; *T_max_* is the maximum temperature; *T_min_* is the room temperature; *Q_s_* is the solvent-related absorbed heat; *I* is the laser power density; and *A_λ_* is the absorbance at 808 nm.

### In vitro catalytic activity of M@GOx-CAT@CuS NPs

The chemodynamic properties of nanoparticles were tested by utilizing 3,3',5,5'-tetramethylbenzidine (TMB) as an indicator. Various concentrations of M@GOx-CAT@CuS NPs (1.5, 3, 6, 12, 25, and 50 μg/ml), H_2_O_2_ (5 mM), and TMB (1 mM) were coincubated for 2 min. Various concentrations of H_2_O_2_ (0, 1.25, 2.5, 5, and 10 mM) were coincubated with M@GOx-CAT@CuS NPs (100 μg/ml) in the presence of indicator at different pH (5 and 6.5) for 30 min. Hydrogen peroxide concentrations (0, 1.25, 2.5, 5, and 10 mM) were coincubated with M@GOx-CAT@CuS NPs (100 μg/ml) for 20 min after 10 min of NIR irradiation. M@GOx-CAT@CuS NPs (100 μg/ml) and H_2_O_2_ (1.25 mM), glucose (5 mM) were incubated for 30 min. M@GOx@CuS NPs (100 μg/ml) were preincubated with different concentrations of glucose solution (1.25, 2.5, 5, and 10 mM) for 30 min. Afterward, each of the reaction solutions were centrifuged (11,000 rpm, 10 min). The absorbance of the supernatants was analyzed using ultraviolet-visible light (UV-Vis, 652 nm).

### Catalytic reaction assay of M@GOx-CAT@CuS NPs

The assay for GOx-catalyzed production of gluconic acid was referenced from previous studies [42]. In short, separate samples that contained equal quantities of GOx were incubated with PBS (pH = 5) containing glucose (50 mM) for 30 min, followed by the reaction of 500 μl of solution A (0.15 mM triethylamine and 5 mM EDTA) and 50 μl of solution B (3 M hydroxylamine [NH_2_OH]) for 25 min. Subsequently, 250 μl of solution C (0.1 M ferric trichloride [FeCl_3_], 1 M HCl, and 0.25 M CCl_3_COOH) was added to the reaction solution, and an enzyme marker measured the absorbance value at 505 nm after 5 min. Five groups of equivalent GOx (GOx, GOx@CuS NPs, GOx-CAT@CuS NPs, M@GOx-CAT@CuS NPs, and M@GOx-CAT@CuS NPs + NIR) were set up to be coincubated with glucose solution (50 mM), and the pH levels in the experimental fluids were recorded at 0, 1, 2, 4, 6, 8, and 12 h. M@GOx-CAT@CuS NPs were added to various concentrations (5, 10, 15, and 20 mM) of glucose solution. Following 1 h of cultivation, centrifugation was carried out at 11,000 rpm for 10 min, and the H_2_O_2_ assay kit was assayed for H_2_O_2_ concentration in the supernatant. H_2_O_2_ (20 mM) solution was coincubated with M@GOx-CAT@CuS NPs [43]. Equivalent GOx concentrations of M@GOx@CuS NPs and M@GOx-CAT@CuS NPs were coincubated with glucose solution, and changes in solution oxygen were detected in real-time using a dissolved oxygen meter, measured every minute for 50 min.

### In vitro cytotoxicity measurement

Mouse breast cancer cells were grown in a 96-well plate with a density of 5 × 10^3^/well for 24 h. CuS NPs (0, 12.5, 25, 50, 100, and 200 μg/ml) were included and cultivated with 4T1 cells either for 12 or 24 h. Incubation with 10% cell counting kit-8 (CCK8) assay reagent (100 μl/well) was performed for 45 min, and cell viability was subsequently quantitated by a microplate reader to record the absorbance at 450 nm.

### Cellular uptake of M@GOx-CAT@CuS NPs

Raw264.7 and 4T1 cells were cultured into confocal dishes and kept in the incubator for 24 h. DiR-labeled NPs (100 μg/ml) were then supplied, and the cells were cultured for 2, 4, and 6 h, respectively. After fixed with 4% paraformaldehyde, nuclei were dyed by 4′, 6-diamidino-2-phenylindole. The cells were inspected with a confocal laser scanning microscope (CLSM). For flow cytometry, cells were trypsin-digested, washed, pelleted with 1,000-rpm centrifugation, and resuspended in PBS.

### Photothermal/chemodynamic/starvation therapies on 4T1 cells

4T1 cells were cultured in 96-well plate for 24 h at a density of 5 × 10^3^/well. M@CuS NPs, M@GOx@CuS NPs, and M@GOx-CAT@CuS NPs (100 μg/ml) were supplemented and 4T1 cells were grown for 4 h. The unabsorbed nanoparticles were removed after washed with PBS. This was followed by laser exposure at 808 nm (1 W/cm^2^, 6 min), and infrared thermograms were taken using CCK8 assay to estimate cell viability.

### In vitro live/dead cell staining and apoptosis assay

4T1 cells were plated at 5 × 10^4^/well and incubated in confocal dishes for 24 h and then treated with differing NPs for 4 h. Upon cleaning the unabsorbed NPs, an 808-nm laser was used to irradiate the cells (1 W/cm ^2^, 6 min). Calcein-AM (2 × 10^−6^ M) and propidium iodide (2 × 10^−6^ M) were added. The cells were then washed twice with fresh medium for CLSM observation. For flow cytometry apoptosis, cells were trypsinized and dyed in an Annexin V-fluorescein isothiocyanate/propidium iodide apoptosis kit.

### Measurement of cellular reactive oxygen species

Detection of intracellular reactive oxygen species (ROS by DCFH-DA probe. Briefly, 4T1 cells were incubated at a density of 5 × 10^4^ in confocal dishes for 24 h. Cells were pretreated with various NPs for 4 h, rinsed 3 times in PBS, and then stained with 10 mM DCFH-DA for 0.4 h before being treated with an 808-nm laser (1 W/cm^2^, 6 min). The treated cells were imaged and evaluated by laser confocal microscopy and flow cytometry.

### Detection of intracellular ATP and H_2_O_2_

4T1 cells were plated in a 6-well plate at a concentration of 4 × 10^5^ cells/well for 24 h. Different groups of nanoparticles were applied and cultivated with cells for varying times. Following 3 rinses in PBS, the cells were illuminated by an 808-nm laser (1 W/cm^2^, 6 min). The cells were collected by centrifugation. Intracellular ATP and H_2_O_2_ levels were assessed based on the protocol of the Enhanced ATP Assay Kit and Hydrogen Peroxide Detection Kit.

### Western blot analysis

Spike 4T1 cells were inoculated into a 6-well plate at a concentration of 4 × 10^5^ cells/well for 24 h. Following a 4-h treatment with varying sets of nanoparticles, the cells were cleaned with PBS and irradiated with 808-nm laser (1 W/cm^2^, 6 min). The treated cells were collected by trypsin digesting. The standard western blot process analyzed the expression of HSP70 and HSP90 proteins.

### Animal models

Female Balb/c mice (6 to 8 wk old) were kindly supplied by the Animal Experiment Center of Chongqing Medical University (Chongqing, China). A total of 1 × 10^6^ 4T1 cells were administered subcutaneously into the second pair of mammary glands of mice in a volume of 100 μl. The size of the tumor growth was observed regularly, and a volume of 50 to 80 mm^3^ was sufficient for therapeutic experiments in vivo.

### In vivo fluorescent and photoacoustic imaging

Tumor-bearing mice (*n* = 3) were treated intravenously with DiR-labeled GOx-CAT@CuS NPs and M@GOx-CAT@CuS NPs diluted with saline (2.5 mg/ml, 200 μl), respectively. Fluorescence imaging of the tumor sites was collected at various intervals (preinjection, 2, 4, 8, 24, 48, and 72 h postinjection), and the fluorescence signals were quantitatively quantified.

For photoacoustic (PA) imaging, after injected with NPs, PA imaging of the tumor region was monitored at various intervals (preinjection, 2, 4, 8, 24, 48, and 72 h postinjection), and PA signals were quantitatively analyzed. M@GOx-CAT@CuS NPs at concentrations of 12.5 to 500 μg/ml were selected for in vitro imaging evaluation.

### Pharmacokinetics and biodistribution of NPs

Tumor-bearing mice were infused intravenously with M@GOx-CAT@CuS (2.5 mg/ml, 200 μl) or an equivalent amount of GOx-CAT@CuS NPs diluted in saline, respectively. Orbital venous plexus blood was obtained at 0.083, 0.5, 1, 2, 4, 8, and 24 h after injection. The copper ion concentration in the blood was assessed using the inductively coupled plasma-optical emission spectrometer (ICP-OES) technique.

To study the biodistribution of M@GOx-CAT@CuS NPs in vivo, mice were injected intravenously with saline-diluted M@GOx-CAT@CuS NPs and GOx-CAT@CuS NPs (2.5 mg/ml, 200 μl). Twenty-four hours later, tumors and major organs (heart, liver, spleen, lungs, and kidneys) were harvested. The copper ion content in the tumors and organs was measured by ICP-OES.

### In vivo photothermal properties of M@GOx-CAT@CuS NPs

Equal amounts of CuS NPs (2.5 mg/ml, 200 μl), M@CuS NPs, M@GOx-CAT@CuS NPs, and saline were individually administered into tumor-bearing mice (*n* = 5). The 808-nm laser (1.5 W/cm^2^, 10 min) was applied after 24 h, and the trend of tumor temperature was noted by infrared thermography.

### In vivo DC cell activation assessment

In vivo immunoactivation of M@GOx-CAT@CuS NPs was evaluated in a mouse 4T1 tumor model. Mice were stratified randomly into 5 groups (saline, M@GOx-CAT@CuS, M@CAT@CuS+NIR, M@GOx@CuS+NIR, and M@GOx-CAT@CuS+NIR) (*n* = 5), when tumor volume approached 50 to 80 mm^3^. NPs (2.5 mg/ml) were injected intravenously on days 0 and 2. On the 18th day, tumors and lymph nodes from mice were obtained for dendritic cells (DC) activation analysis. Tumor cell suspensions were labeled by fluorescein isothiocyanate anti-CD11c, PE anti-CD80, and APC anti-CD86 for flow cytometry analysis [44].

### In vivo efficacy of combined therapy

As tumor sizes approached 50 to 80 mm^3^, the mice were randomized into 5 groups (*n* = 5): saline, M@GOx-CAT@CuS, M@CAT@CuS + NIR, M@GOx@CuS + NIR, and M@GOx-CAT@CuS + NIR. NPs (2.5 mg/ml) were infused intravenously on day 0. After 24 h, 808-nm laser treatment (1.5 W/cm^2^, 10 min) was performed. Dosing was carried out on day 2, and a second laser irradiation was performed on day 3. Tumors and mouse body weight were monitored at intervals of 1 d. The tumor size (V) was identified according to the given formula: *V* = (width^2^ × length)/2. At the end of treatment (day 18), tumors and organs were harvested for staining observation.

### In vivo biosafety assessment

Randomly distributed Balb/c mice into 5 groups (*n* = 5) and infused i.v. with saline-diluted M@GOx-CAT@CuS NPs (2.5 mg/ml). Blood and major organs (heart, liver, spleen, lungs, and kidneys) were obtained at 1, 7, 14, and 28 d after injection. Blood was subjected to routine hematology and serum biochemistry analysis. The major organs were placed in tissue fixative for 24 h. Then, they were dewatered and dipped in wax and placed in an embedding machine for embedding. Paraffin sections were deparaffinized to water. Sections were stained into hematoxylin dye for 3 to 5 min, rinsed repeatedly, then sequentially put into 85% and 95% graded alcohol dehydration for 5 min and into eosin dye for 5 min. In sequence, the sections were put into anhydrous ethanol, dimethyl, and xylene. After sealed by neutral tree glue, the slices were observed by a microscope.

### Statistical analysis

All data were presented as mean ± SD and analyzed using GraphPad Prism 7.0. To assess the significant difference between different groups, 1-way analysis of variance (ANOVA), 2-way ANOVA, and *t* test statistical analysis were applied. Data were categorized according to *P* value and deemed statistically meaningful at ^*^P < 0.05, ^**^P < 0.01, ^***^P < 0.001, and ^****^P < 0.0001.

## Results and Discussion

### Characterization of the CuS NPs and M@GOx-CAT@CuS NPs

**Fig. 2.**
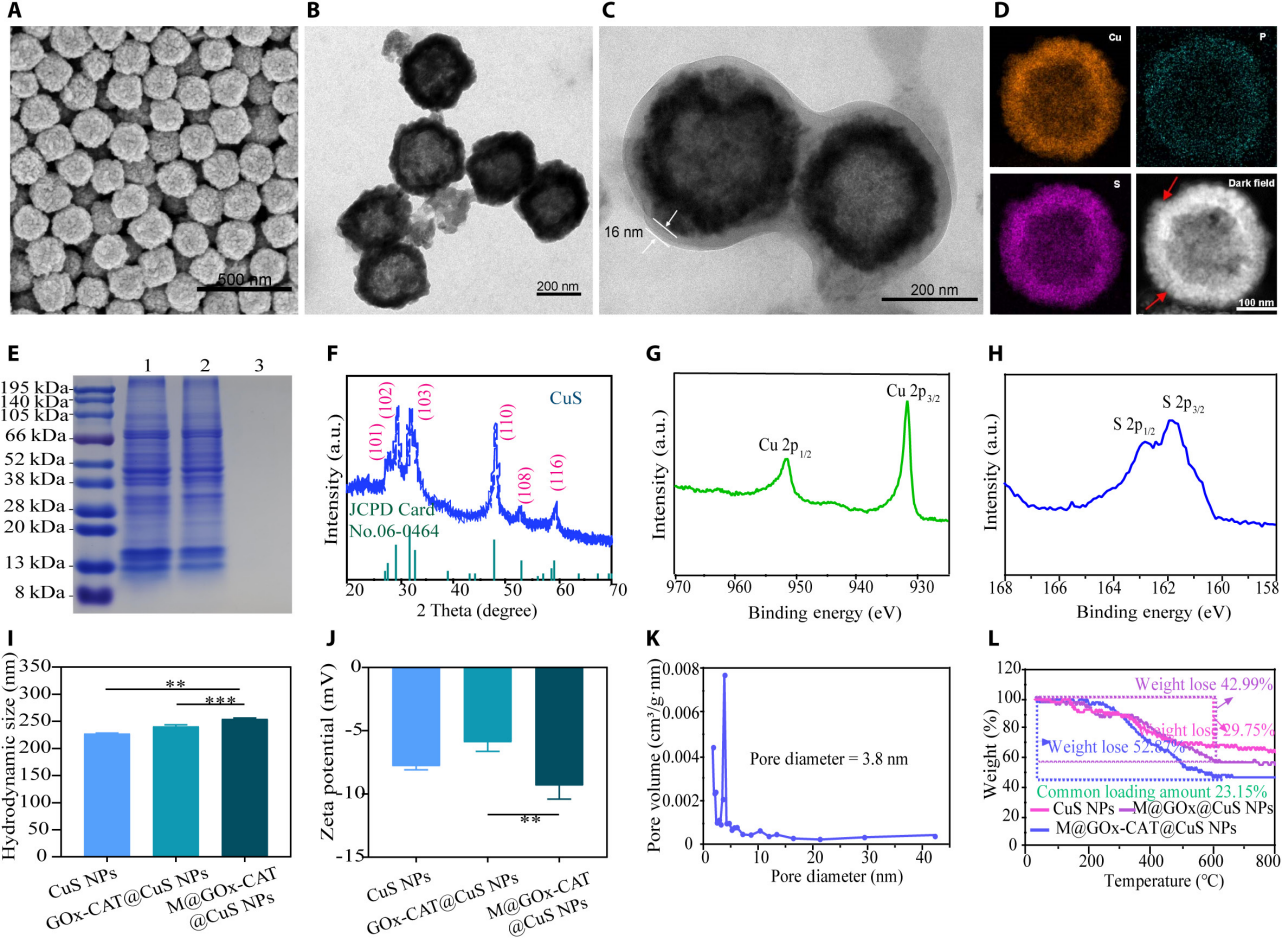
Characterization of M@GOx-CAT@CuS NPs. (A) Scanning electron microscopy image of CuS NPs. Scale bar: 500 nm. (B and C) Transmission electron microscopy images of CuS NPs and M@GOx-CAT@CuS NPs. Scale bar: 200 nm. (D) Elemental distribution mappings of M@GOx-CAT@CuS NPs (the red arrow indicates the cell membrane). Scale bar: 100 nm. (E) Sodium dodecyl sulfate polyacrylamide gel electrophoresis analysis of different samples (1: macrophage membrane, 2: M@CuS NPs, 3: CuS NPs). (F) X-ray diffraction pattern of CuS NPs. (G and H) XPS spectra of CuS NPs. (I and J) Hydrodynamic diameters and zeta potentials of CuS NPs, GOx-CAT@CuS NPs, and M@GOx-CAT@CuS NPs. (K) Pore size distribution of CuS NPs. (L) TGA traces of CuS NPs, M@GOx@CuS NPs, and M@GOx-CAT@CuS NPs. Significant difference was regarded as ^**^P < 0.01, ^***^P < 0.001.

The CuS NPs with hollow mesoporous structures were synthesized by template sacrifice method [45]. Two enzymes GOx and CAT were successfully coloaded into the CuS NPs. M@GOx-CAT@CuS NPs were obtained after encapsulating macrophage membranes (M) on the surface of the nanoparticles. Transmission electron microscopy and scanning electron microscopy findings revealed that the CuS NPs were spherical, regular grains, and hollow structures (Fig. 2A and B). The core–shell structure of M@GOx-CAT@CuS NPs was also observed by electron microscopy (Fig. 2C), indicating that the nanoparticles were successfully coated by the macrophage membranes, which were roughly 16 nm thick. The elemental distribution mappings of M@GOx-CAT@CuS NPs were displayed in Fig. 2D. The results indicated the presence of P elements in the phospholipid molecules on the surface of the nanoparticles, confirming the successful wrapping of the macrophage membrane. In addition, the nanoparticles were studied by sodium dodecyl sulfate polyacrylamide gel electrophoresis. The occurrence of macrophage membrane wrapping on the surface of nanoparticles was observed (Fig. 2E). These results showed that the M@GOx-CAT@CuS NPs had the same characteristics as macrophage membranes, indicating that the cell membrane had been successfully encapsulated on the NPs surface. The phase structure of the hollow spheres was further examined using x-ray diffraction. The sample’s peaks align with the CuS crystal’s crystal faces (JCPD 06-0464), as seen in Fig. 2F. The manufactured CuS NPs displayed superb crystal structure and excellent purity in accordance with the findings. The Cu and S chemistry valency states were analyzed by x-ray photoelectron spectroscopy (XPS), in which high-resolution Cu 2p XPS patterns at 931.7 and 951.8 eV were characterized by the binding energies of the feature peaks of Cu 2p_3/2_ and Cu 2p_1/2_, respectively. The S 2p XPS profile’s binding energies, which were positioned at 161.9 and 162.8 eV, respectively, were the signature peaks of S 2p_3/2_ and S 2p_1/2_ (Fig. 2G and H). The results demonstrated the occurrence of sulfides or disulfides in the synthetics. The hydrodynamic average particle size of the hollow CuS NPs was estimated to be 227.4 nm based on dynamic light scattering measurements. The hydrodynamic size of the GOx-CAT@CuS (240.3 nm) and M@GOx-CAT@CuS NPs (254.1 nm) was increased by 12.9 and 26.7 nm, respectively, when compared to the uncoated CuS (Fig. 2I and J). Additionally, M@GOx-CAT@CuS NPs suspended in PBS for 7 d exhibited high stability(Fig. S1). As shown in Fig. S2, CuS NPs showed apparent absorption characteristics in the NIR region, and the absorption capacity gradually enhanced with increasing concentration. N_2_ adsorption–desorption experiments showed that CuS NPs were hollow mesoporous structures with a specific surface area of 11 m^2^/g (Fig. S3) and an average pore size of 3.8 nm (Fig. 2K), which was favorable for the effective loading of enzymes. Based on the bovine serum albumin standard curve (Fig. S4), the encapsulation rate of GOx and CAT was 87.39%, and its high encapsulation rate not only improved the drug utilization but also prolonged the drug release time. The TGA showed that the payload of these enzymes was 23.15% (Fig. 2L), with the encapsulation rate of 90.84%. The TGA results were in general concordance with the BCA results obtained.

### In vitro photothermal and enzymatic chemokinetics of M@GOx-CAT@CuS NPs

**Fig. 3.**
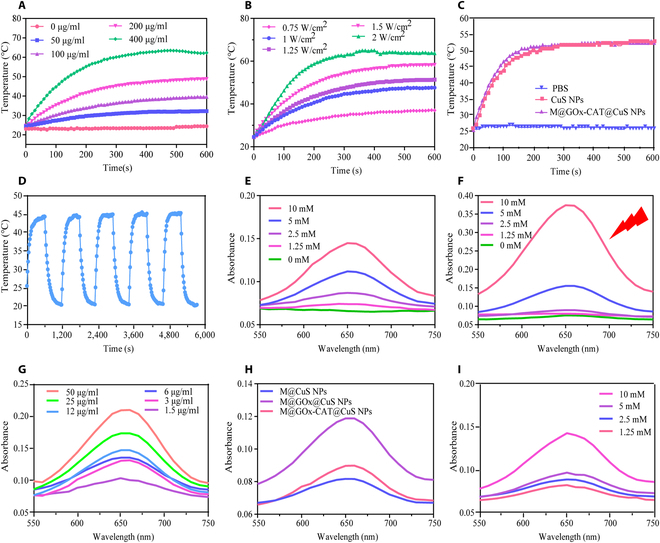
In vitro photothermal and catalytic properties of M@GOx-CAT@CuS NPs. (A) Time–temperature curves for different concentrations of M@GOx-CAT@CuS NPs (1 W/cm^2^, 10 min). (B) Time–temperature curves of M@GOx-CAT@CuS NPs at different 808-nm laser power densities (200 μg/ml). (C) Time–temperature curves of PBS, CuS NPs, and M@GOx-CAT@CuS NPs (200 μg/ml) under irradiation with laser power of 1.25 W/cm^2^. (D) Time–temperature curves of M@GOx-CAT@CuS NPs (200 μg/ml) under 5 repeated heating and cooling cycles (1 W/cm^2^). (E and F) UV-Vis absorption spectra of TMB oxidation catalyzed by different concentrations of H_2_O_2_ and M@GOx-CAT@CuS NPs (100 μg/ml) in the absence or presence of laser irradiation (pH = 6.5, reaction time: 30 min). (G) UV-Vis absorption spectra of TMB oxidation catalyzed by different concentrations of M@GOx-CAT@CuS NPs and H_2_O_2_ (5 mM) (reaction time: 2 min). (H) UV-Vis absorption spectra of TMB oxidation catalyzed by M@CuS NPs, M@GOx@CuS NPs, and M@GOx-CAT@CuS NPs (100 μg/ml) in the presence of H_2_O_2_ (1.25 mM) and glucose (5 mM) (reaction time: 30 min). (I) UV-Vis absorption spectra of TMB oxidation catalyzed by M@GOx@CuS NPs in the presence of different concentrations of glucose solutions (reaction time: 30 min).

The photothermal properties and enzymatic chemokinetics of M@GOx-CAT@CuS NPs were evaluated. Figure 3A and Fig. S5 showed a gradual increase in the solution temperature as concentration and time increased. As the dose increased to 400 μg/ml, the temperature of M@GOx-CAT@CuS NPs increased from 24.4 to 62.3 °C upon 808-nm laser exposure. For 200 μg/ml M@GOx-CAT@CuS NPs, an increasing trend of temperature rise with increasing power density was observed (Fig. 3B and Fig. S6). The temperature of M@GOx-CAT@CuS NPs was elevated from 24.6 to 63.8 °C under 808-nm laser output of 2 W/cm^2^. The temperature–time curves of M@GOx-CAT@CuS NPs and CuS NPs were in close agreement, indicating that drug loading and macrophage membrane encapsulation did not affect the heat production effect of NPs (Fig. 3C). The photothermal stability of M@GOx-CAT@CuS NPs (Fig. 3D) was verified by following 5 heating and cooling cycles. The photothermal conversion efficiency of M@GOx-CAT@CuS NPs was calculated as 26.42% through Figs. S7 and S8. Such results indicated that CuS NPs exhibited excellent photothermal conversion ability and photothermal stability [46].

In this study, TMB was used as an indicator to verify the chemodynamic properties of the fabricated nanoparticles. In the presence of oxidants like ·OH, TMB is oxidized to blue oxTMB with a large absorbance value at 652 nm [47]. The Michaelis–Menten constant (*K*_m_) and the maximum initial velocity (*V*_m_) were obtained using the Lineweaver–Burk equation as shown in Figs. S9 and S10. The *K*_m_ value indicates the affinity of the substrates (TMB and H_2_O_2_) for the catalyst, and the lower the *K*_m_ value, the stronger the affinity. The *K*_m_ values of the M@GOx-CAT@CuS NPs with H_2_O_2_ and TMB as substrates were 1.13 and 0.22 mM, respectively, indicating that M@GOx-CAT@CuS had a relatively high affinity for the substrate. The absorbance of TMB catalyzed by M@GOx-CAT@CuS NPs subjected to laser irradiation (Fig. 3F) was higher than those of the nonlaser-irradiated group (Fig. 3E) at the same concentration. The results indicated that the temperature increase under laser irradiation was favorable for promoting CDT. Furthermore, the absorbance of M@GOx-CAT@CuS NPs at 652 nm increased with increasing H_2_O_2_ concentration, regardless of laser irradiation. Figure 3G demonstrated that the catalytic ability of M@GOx-CAT@CuS NPs to produce ·OH was enhanced with increasing nanoparticle concentration. Moreover, the absorbance values of different groups of reaction solutions were measured at 652 nm, and it was found that M@GOx-CAT@CuS could only perform CDT to generate ROS in the presence of glucose (Fig. S11). Meanwhile, the study recorded the UV-Vis absorption spectra of M@GOx-CAT@CuS and glucose at different times, and the results showed that, with laser irradiation and the prolongation of the coincubation time, the efficiency of M@GOx-CAT@CuS catalyzed the production of H_2_O_2_ from glucose to generate CDT with a gradual increase in efficiency, suggesting that the increase in temperature increased the activity of GOx enzyme as well as the efficiency of the production of ROS (Fig. S11B and C). The highest absorbance of M@GOx@CuS NPs was observed at 652 nm in glucose solution, suggesting that the GOx-catalyzed H_2_O_2_ generation facilitated the CDT (Fig. 3H). Moreover, increasing the glucose concentration (from 1.25 to 10 mM) accelerated the catalyzed TMB oxidation due to the continuous production of H_2_O_2_ and the continuous decrease in pH (Fig. 3I). The catalytic effect of M@GOx-CAT@CuS NPs was more marked at pH = 5 (Fig. S12). This indicated that tumor acidic environment was favorable for CDT.

### In vitro enzyme cascade reactions and enzyme stability

In this study, a NIR-triggered dual enzyme cascade reaction nanoplatform was constructed to integrate multiple therapies to boost the efficacy of tumor treatments. Figure 4A illustrated the mechanism of cascade catalytic therapy, where GOx catalyzed the transformation of glucose into gluconate and H_2_O_2_, facilitating starvation therapy of cancer. GOx products (H_2_O_2_) played an essential role in the cascade reaction. On the one hand, CuS NPs catalyze hydrogen peroxide intermediated to generate toxic ·OH, which killed tumor cells. On the other hand, CAT could catalyze hydrogen peroxide intermediates to generate O_2_, which further accelerated the oxidation of GOx.

**Fig. 4.**
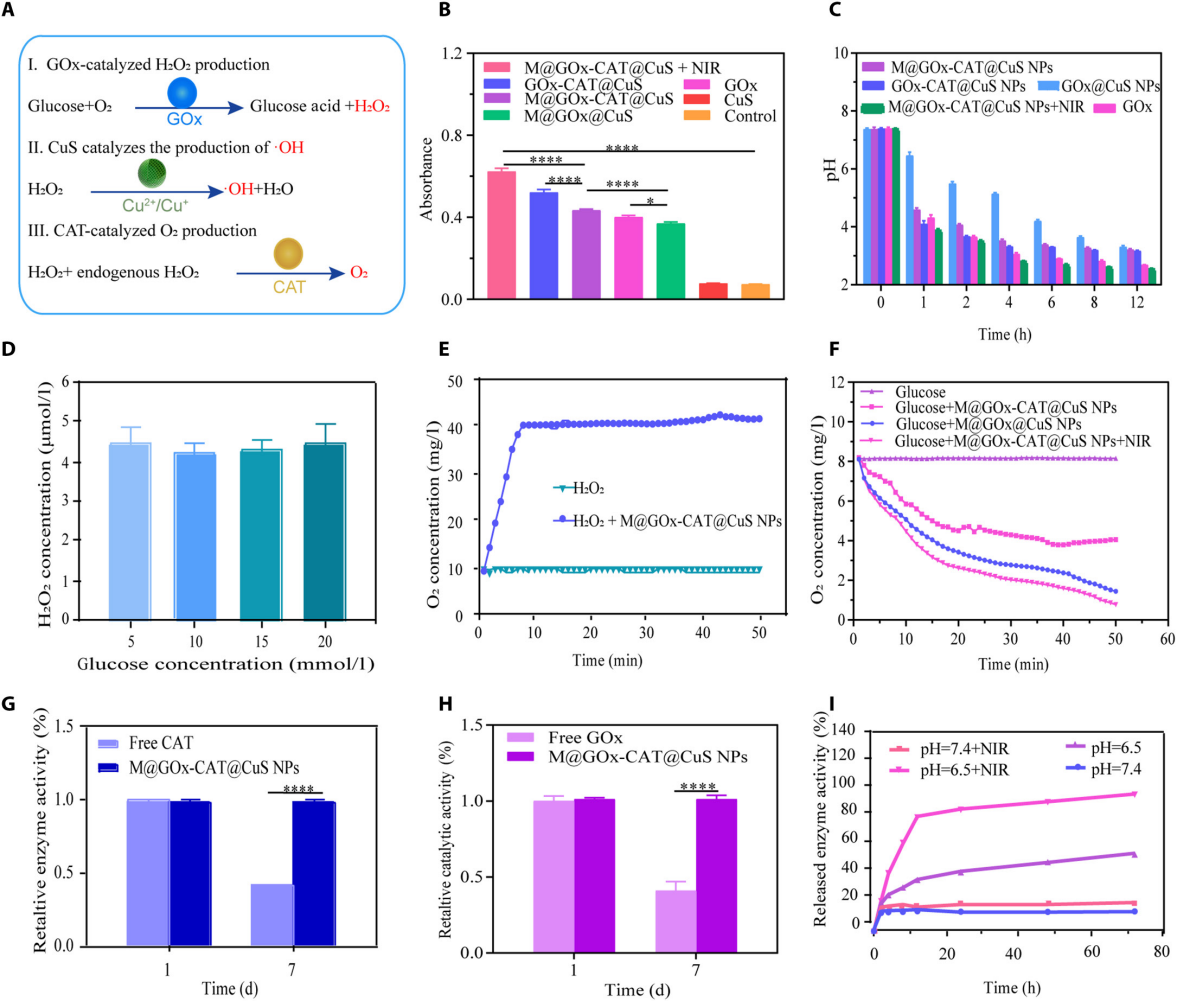
Cascade catalyzed reaction of M@GOx-CAT@CuS NPs. (A) Schematic diagram of the cascade catalytic process of M@GOx-CAT@CuS NPs. (B) Absorbance values at 505 nm for different samples in the determination of gluconic acid (mean ± SD, *n* = 3). (C) The changes in pH of different samples in glucose solution (50 mM) with time (mean ± SD, *n* = 3). (D) The H_2_O_2_ concentration of M@GOx-CAT@CuS NPs incubated for 1 h in the presence of different concentrations of glucose solutions (mean ± SD, *n* = 3). (E and F) The variations of oxygen concentration with time in H_2_O_2_ and glucose solution solution for different samples. (G and H) The relative enzyme activity of CAT and GOx of different samples at day 1 and day 7 (mean ± SD, *n* = 3). (I) Dual enzyme cumulative release rates of M@GOx-CAT@CuS NPs under different conditions. Significant difference was regarded as ^*^P < 0.05, ^****^P < 0.0001.

In the detection of gluconic acid, the catalytic product of GOx, gluconic acid reacts with other mixtures to form a reddish compound with a characteristic absorption peak at 505 nm. In Fig. 4B, the M@GOx-CAT@CuS group produced more gluconic acid than the M@GOx@CuS group, suggesting that the oxygen provided by CAT-catalyzed H_2_O_2_ facilitated GOx catalysis, and the M@GOx-CAT@CuS NPs group produced the most gluconic acid under laser irradiation, indicating that elevated temperatures increased GOx enzymatic activity. In addition, the highest degree of pH reduction was observed in the M@GOx-CAT@CuS NPs + NIR group by measuring the pH of different groups of nanoparticles (Fig. 4C), which was consistent with the results in Fig. 4B. Additionally, the H_2_O_2_ concentration of M@GOx-CAT@CuS NPs in glucose solution was not dramatically changed with increasing glucose concentration (Fig. 4D). Although the elevated glucose concentration supplied GOx with sufficient feedstock to produce H_2_O_2_, the CAT encapsulated in the NPs continuously decomposed the generated H_2_O_2_, resulting in a relatively constant H_2_O_2_ concentration. For further verification of the catalytic activity of CAT, the dissolved oxygen fraction in H_2_O_2_ solutions was recorded (Fig. 4E). The oxygen concentration in the H_2_O_2_ alone group barely changed, while that in the M@GOx-CAT@CuS NPs + H_2_O_2_ group increased from 9.11 mg/l to 41.54 mg/l. This suggested that the activity of CAT encapsulated in CuS NPs was not affected and could catalyze the generation of O_2_ from H_2_O_2_. M@GOx@CuS NPs-mediated glucose oxidation decreased the oxygen concentration from 8.12 mg/l at the outset to 1.44 mg/l. By contrast, M@GOx-CAT@CuS NPs slowed the decrease in dissolved oxygen levels in glucose solution (Fig. 4F). It could be a result of CAT catalyzing the production of O_2_ from H_2_O_2_, a product of GOx, further evaluating the enzymatic cascade reaction between GOx and CAT. However, the M@GOx-CAT@CuS NPs + NIR group showed the highest decrease in oxygen concentration, probably due to the increase in temperature and thus the increase in GOx enzyme activity, whereas the CAT enzyme activity remained stable and the rate of oxygen consumption was much higher than the rate of oxygen production. Since both GOx and CAT were enzymes, there was a risk of loss of their activity. Compared with the activity of free CAT and GOx, the CAT and GOx activity of M@GOx-CAT@CuS NPs was essentially unchanged (Fig. 4G and H), indicating that CuS protective shell protected the enzyme activity and considerably prolonged the stable condition of the enzyme.

We investigated the enzyme activity under different temperatures. The results showed the GOx enzyme activity increased at 45 °C after incubation, whereas the activity of CAT decreased slightly (Fig. S13). Following BCA concentration determination (Fig. S14), the cumulative release of the enzyme reached 54.47% at 72 h in an acidic environment, whereas in a neutral environment, the cumulative release of the enzyme was only 13.87%. Upon laser irradiation, the cumulative release rate of the enzyme was improved in a neutral environment and considerably increased in an acidic environment, suggesting that the CuS NPs had NIR and pH-responsive properties (Fig. 4I).

### In vitro tumor targeting ability of M@GOx-CAT@CuS NPs

Integrin α4β1 on macrophage membranes interacts with the VCAM-1 on mammary cancer cells to increase the affinity between macrophages and cancer cells, allowing the bionic nanodrugs to actively target the tumor site and prolong the circulation time of nanodrugs in vivo [48]. As shown in Fig. 5A and B, the uptake of M@GOx-CAT@CuS NPs by 4T1 cells was higher than that of GOx-CAT@CuS NPs at 2, 4, and 6 h, respectively. It suggested that M@GOx-CAT@CuS NPs had the capability to target tumor cells. However, macrophage immunofluorescence showed the reverse result (Fig. 5C and D), suggesting that M@GOx-CAT@CuS NPs could evade phagocytosis by the endothelial system. Moreover, the outcomes of the flow cytometry assay also illustrated that 4T1 cell uptake of M@GOx-CAT@CuS NPs was considerably higher compared to the uncoated group (Fig. 5E). These findings indicated that macrophage membrane-encapsulated mimetic NPs could escape phagocytosis by the endothelial reticular system and actively target the tumor site.

**Fig. 4.**
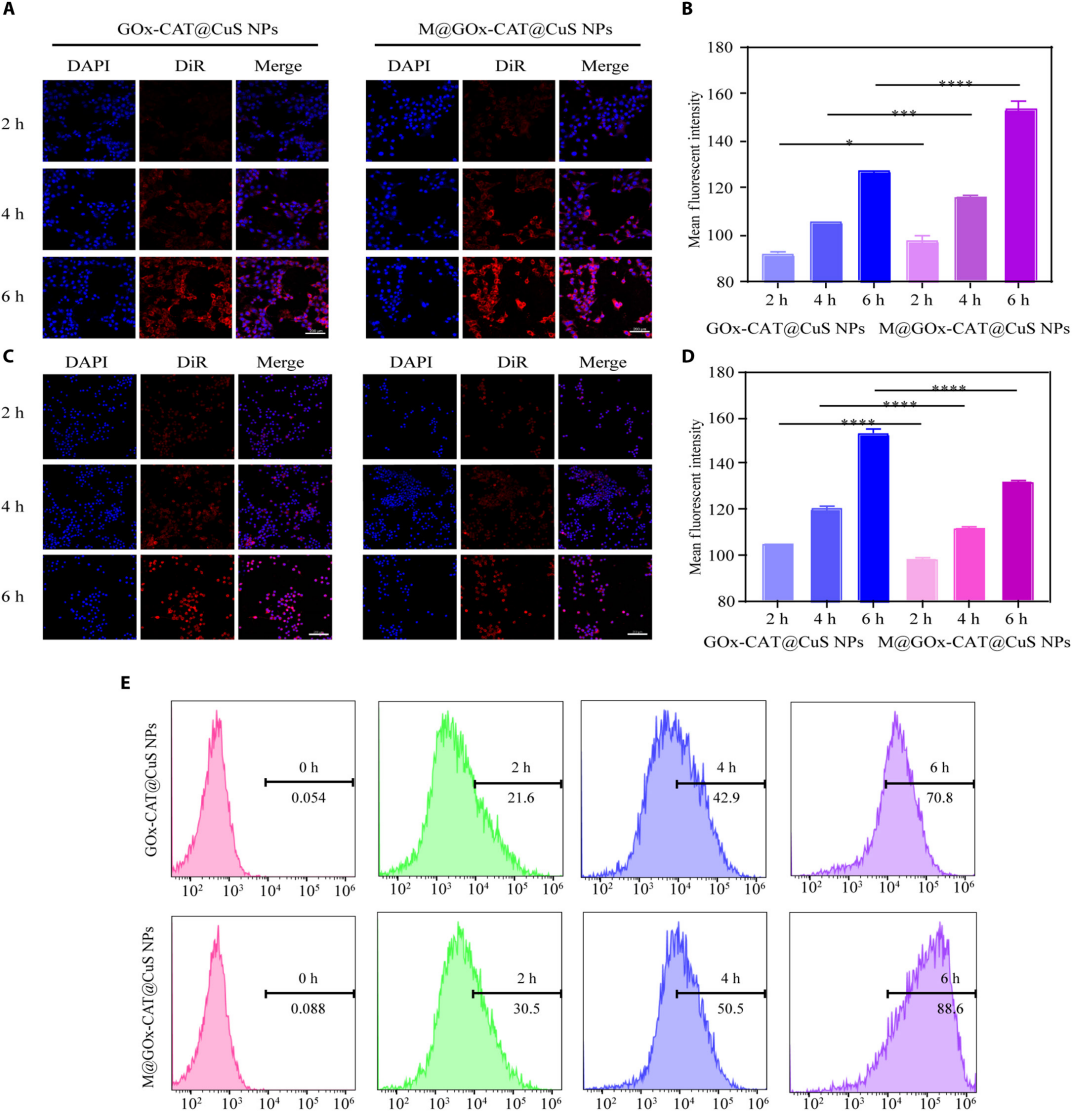
Targeted recognition of 4T1 cells by M@GOx-CAT@CuS NPs. (A) The CLSM images and (B) mean fluorescence intensity of GOx-CAT@CuS NPs and M@GOx-CAT@CuS NPs incubated with 4T1 cells for 2, 4, and 6 h, respectively (mean ± SD, *n* = 3). (C) The CLSM images and (D) mean fluorescence intensity of GOx-CAT@CuS NPs and M@GOx-CAT@CuS NPs incubated with macrophages for 2, 4, and 6 h, respectively (mean ± SD, *n* = 3). (E) Flow cytometric assays of GOx-CAT@CuS NPs and M@GOx-CAT@CuS NPs with different times of 4T1 cell culture. Significant difference is regarded as ^*^P < 0.05, ^***^P < 0.001,^****^P < 0.0001.

### Intracellular cascade catalytic reaction and cytotoxic apoptosis assay

**Fig. 6.**
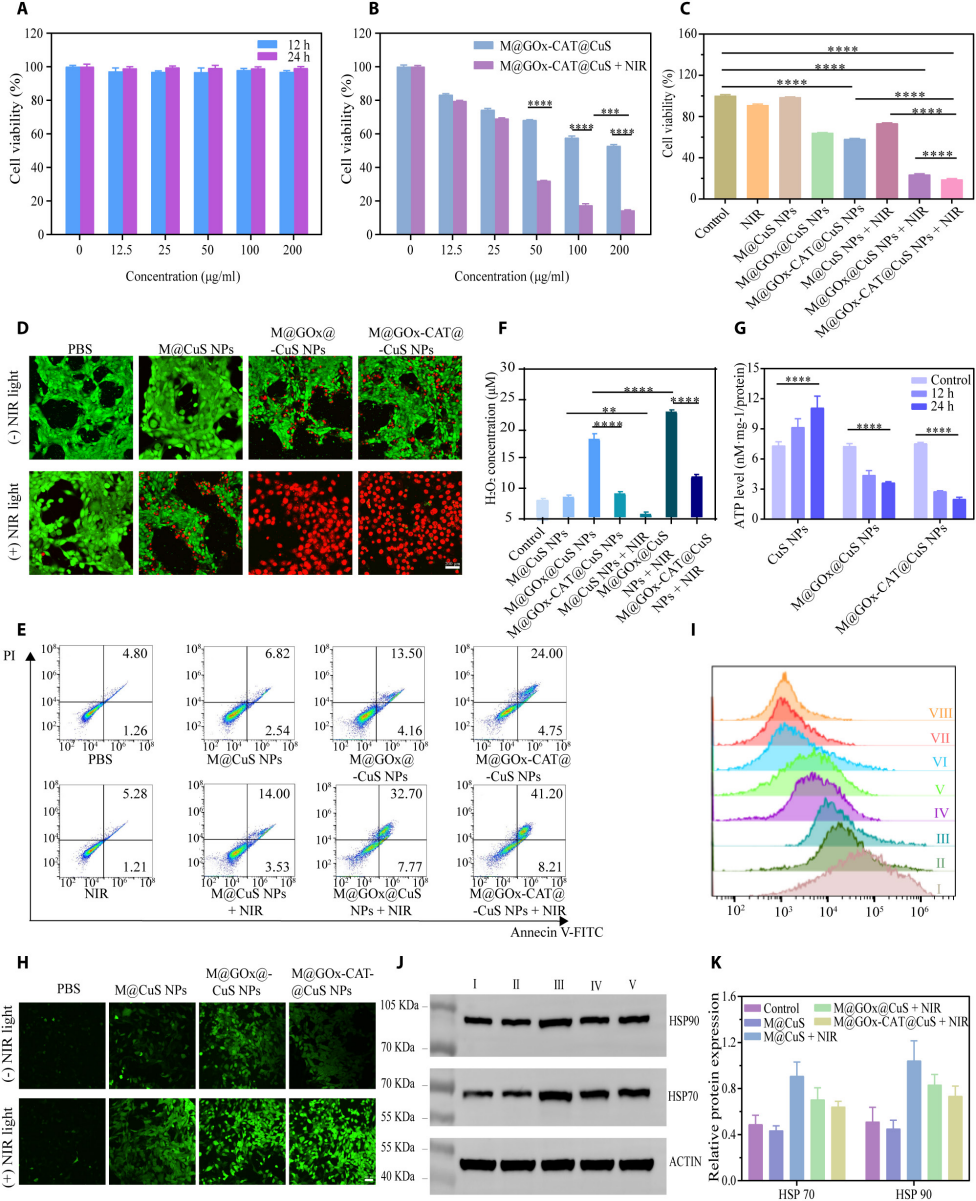
Synergistic antitumor effects on 4T1 cells in vitro. (A) Relative cell viability of 4T1 cells treated with different concentrations of CuS NPs for 12 and 24 h (mean ± SD, *n* = 3). (B) Relative cell viability of various concentrations of M@GOx-CAT@CuS NPs incubated with 4T1 cells for 4 h with or without laser irradiation (mean ± SD, *n* = 3). (C) Relative activity of 4T1 cells after treatment with different groups of NPs (100 μg/ml) for 4 h (mean ± SD, *n* = 3). (D) Representative CLSM images and (E) flow cytometry profiles of 4T1 cells after different treatments. Scale bar: 200 μm. (F) The H_2_O_2_ concentration in 4T1 cells after different treatments (mean ± SD, *n* = 3). (G) Intracellular ATP levels in CuS NPs, M@GOx@CuS NPs, and M@GOx-CAT@CuS NPs incubated with 4T1 cells for 12 and 24 h (mean ± SD, *n* = 3). (H) Representative CLSM images and (I) flow cytometry profiles of DCFH-DA-stained 4T1 cells after different treatments. Scale bar: 200 μm. (I: PBS, II: NIR, III: M@GOx-CAT@CuS NPs, IV: M@CuS NPs, V: M@GOx@CuS NPs, VI: M@CuS NPs + NIR, VII: M@GOx@CuS NPs + NIR, VIII: M@GOx-CAT@CuS NPs + NIR). (J and K) The expression levels of HSP90 and HSP70 were detected by western blotting after treating cells in different groups (I: blank control, II: M@CuS NP, III: M@CuS NPs + NIR, IV: M@COx@CuS NPs + NIR, V: M@COx-CAT@CuS NPs + NIR). Significant difference is regarded as ^**^P < 0.01, ^***^ *P* < 0.001,^****^P < 0.0001.

Evaluation of the cytotoxic and therapeutic effects of different nanoparticles was conducted using CCK8 cell viability tests. After the coincubation of CuS NPs with 4T1 cells, the viability of 4T1 cells was not markedly reduced even after the dose was increased to 200 μg/ml (Fig. 6A). The results demonstrated that CuS was not appreciably toxic to 4T1 cells and was biocompatible. We evaluated the cellular activity of CuS NPs after incubation with 4T1 cells at different power densities (6 min) in order to select the appropriate laser power density for subsequent cell therapy (Fig. S15). It was found that at different concentrations of CuS NPs, the cellular activity at 1 W/cm^2^ was higher than that at 1.75 W/cm^2^. Thus, the 1 W/cm^2^ power density was selected for subsequent cell experiments. We evaluated the 4T1 cell activity of varying concentrations of M@GOx-CAT@CuS NPs on 808-nm laser treatment (1 W/cm^2^, 6 min) (Fig. 6B). It showed that the cellular activity of the M@GOx-CAT@CuS NPs at 200 and 100 μg/ml concentrations was of 14.32% and 17.46%, respectively. The cell viability of M@GOx-CAT@CuS NPs (100 μg/ml) was relatively high. Hence, M@GOx-CAT@CuS NPs of 100 μg/ml were chosen for the subsequent cellular experiments. These results indicated an increase in cytotoxicity with increasing M@GOx-CAT@CuS NPs concentration and laser irradiation power. The combined treatment of M@GOx-CAT@CuS NPs and laser radiation showed a notable increase in cytotoxicity and an 81.14% decrease in cellular activity as compared to M@CuS NPs alone (Fig. 6C). In Fig. S16, we observed the warming effect of different groups of nanoparticles and found that the temperature level of cell exposure was always below 45 °C, stimulating cell starvation and CDT synergistic treatment through a mild photothermal effect.

To further confirm that the designed enzymatic cascade reaction system exhibited enhanced synergistic therapeutic effects, we validated these findings using staining of live and dead cells as well as flow analysis of apoptosis. Green fluorescent indicated living cells and red fluorescent indicated dying cells. The synergistic treatment group M@GOx-CAT@CuS NPs + NIR exhibited almost exclusively red fluorescence, whereas green fluorescence was mainly present in the control, NIR, and M@CuS NPs groups (Fig. 6D), which was consistent with the cytotoxicity findings. Flow cytometry results indicated that M@GOx-CAT@CuS NPs induced 49.41% apoptosis under NIR, which was markedly higher than the 17.53% and 28.75% apoptosis after treatment of cells with M@CuS NPs + NIR and M@GOx-CAT@CuS NPs (Fig. 6E).

We evaluated intracellular H_2_O_2_ levels measured by the H_2_O_2_ Assay Kit. GOx-mediated glucose oxidation in M@GOx@CuS produced large amounts of H_2_O_2_ compared to endogenous H_2_O_2_ produced by the PBS group. However, the introduction of CAT into the nanoplatforms reduced the intracellular levels of H_2_O_2_ because CAT degraded intracellular H_2_O_2_ (Fig. 6F). After laser irradiation, the H_2_O_2_ level decreased in the M@CuS + NIR group indicating that temperature promoted CDT. The highest intracellular H_2_O_2_ level was found in the M@GOx@CuS + NIR group, indicating that CuS-mediated mild photothermal heat led to increased activity of GOx, which promoted the catalytic effect of GOx. Intracellular ATP was measured following different treatments to assess the effect of GOx-mediated starvation treatment upon ATP formation (Fig. 6G). Compared with PBS-treated cells, the ATP level of M@GOx-CAT@CuS NPs was reduced by 63.31% and 73.51% at 12 and 24 h, respectively. These effects suggested that glucose depletion via GOx could lower intracellular ATP levels by disrupting the energy pathway and that the cascade reaction between GOx and CAT could exacerbate glucose depletion.

DCFH-DA produces green fluorescence by ROS oxidation and was therefore used to assess ROS production in 4T1 cells. As illustrated in Fig. 6H and Fig. S17, M@CuS NPs-treated 4T1 cells could emit a weaker green fluorescent signal compared with the control and NIR-only groups. This was possibly due to the fact that the overexpressed H_2_O_2_ in the tumor cells could produce a small amount of ·OH with Cu^2+^/Cu^+^ through a Fenton-like reaction. M@GOx@CuS NPs exhibited strong green fluorescence due to H_2_O_2_ production. Moreover, the rate of H_2_O_2_ production was accelerated by CAT providing O_2_ for GOx oxidation under NIR, resulting in an increasing fluorescence intensity. Flow cytometry also confirmed these findings (Fig. 6I). To investigate the promotion mechanism of starvation treatment on mPTT, we detected the levels of HSP70 and HSP90 expression in 4T1 cells following different treatments by western blotting. As illustrated in Fig. 6J and K, M@GOx-CAT@CuS NPs remarkably reduced intracellular HSP70 and HSP90 expression through a cascade reaction. These correlated with trends in intracellular ATP expression levels (Fig. 6G). The above results demonstrated that starvation treatment effectively inhibited ATP expression and further down-regulated HSP, thus reducing cellular heat resistance.

### In vivo biodistribution and tumor-targeted M@GOx-CAT@CuS NPs

**Fig. 7.**
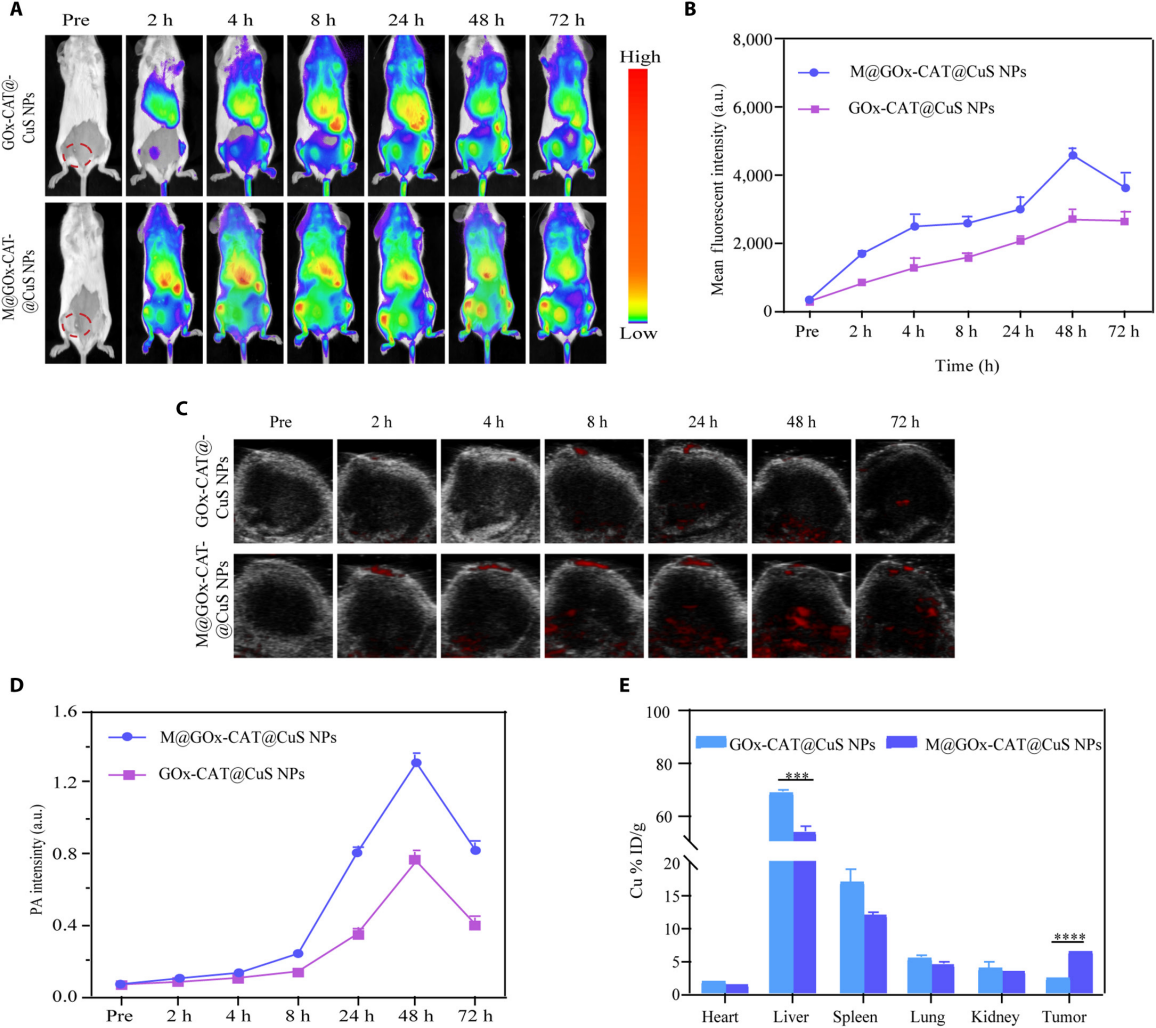
In vivo biodistribution and tumor targeting ability of M@COx-CAT@CuS NPs. (A and B) In vivo fluorescence imaging and analysis of fluorescence intensity at different time points after intravenous injection of GOx-CAT@CuS NPs and M@GOx-CAT@CuS NPs (2.5 mg/ml, 200 μl) in tumor-bearing mice (mean ± SD, *n* = 3). (C and D) In vivo photoacoustic imaging and PA intensity analysis at different time points after intravenous injection of GOx-CAT@CuS NPs and M@GOx-CAT@CuS NPs (2.5 mg/ml, 200 μl) in tumor-bearing mice (mean ± SD, *n* = 3). (E) In vivo biodistribution of GOx-CAT@CuS NPs and M@GOx-CAT@CuS NPs assessed by ICP-OES (mean ± SD, *n* = 3). Significant difference is regarded as ^***^ *P* < 0.001,^****^P < 0.0001.

Motivated by the in vitro cell-targeting ability (Fig. 5), we studied the biodistribution of M@GOx-CAT@CuS NPs in tumor-bearing mice. As shown in Fig. 7A and B, DiR-labeled M@GOx-CAT@CuS NPs and GOx-CAT@CuS NPs were found to be accumulated in the tumor area and reached a maximum at 48 h after injection. In particular, the fluorescence intensity of DiR-labeled M@GOx-CAT@CuS NPs aggregated in the tumor region was 1.7 fold greater compared to GOx-CAT@CuS NPs. Moreover, we performed ex vivo fluorescence imaging (Fig. S18). M@GOx-CAT@CuS NPs mainly accumulated in the tumor region, whereas GOx-CAT@CuS NPs accumulated relatively low in the tumor and stayed mainly in the liver and spleen. The above results indicated that M@GOx-CAT@CuS NPs could escape phagocytosis by the endothelial reticular system and target the tumor site in mice.

CuS NPs are commonly applied as photoacoustic imaging contrast agents due to their excellent NIR absorption and photothermal conversion [49]. Consequently, we investigated in vitro as well as in vivo photoacoustic images of M@GOx-CAT@CuS NPs. The photoacoustic signal intensity showed a linear increase with growing concentrations of M@GOx-CAT@CuS NPs (Fig. S19). Figure 7C and D shows that the PA signal values of M@GOx-CAT@CuS NPs were higher than those of GOx-CAT@CuS NPs at the tumor site at various moments, and the strongest PA signal intensity was reached at 48 h, which was concordant with the fluorescent images findings (Fig. 7A and B).

Additionally, to explore the biodistribution of NPs, copper ion levels in major organs (heart, liver, spleen, lung, and kidney) as well as tumors were assessed by ICP-OES 24 h following intravenous infusion of M@GOx-CAT@CuS NPs and GOx-CAT@CuS NPs (Fig. 7E). The above results demonstrated that the macrophage membrane envelope was able to target the tumor tissue and reduce the accumulation in the liver, which was consistent with the fluorescence imaging (Fig. 7A and B) and photoacoustic imaging results (Fig. 7C and D).

### Synergistic antitumor efficacy in vivo

**Fig. 8.**
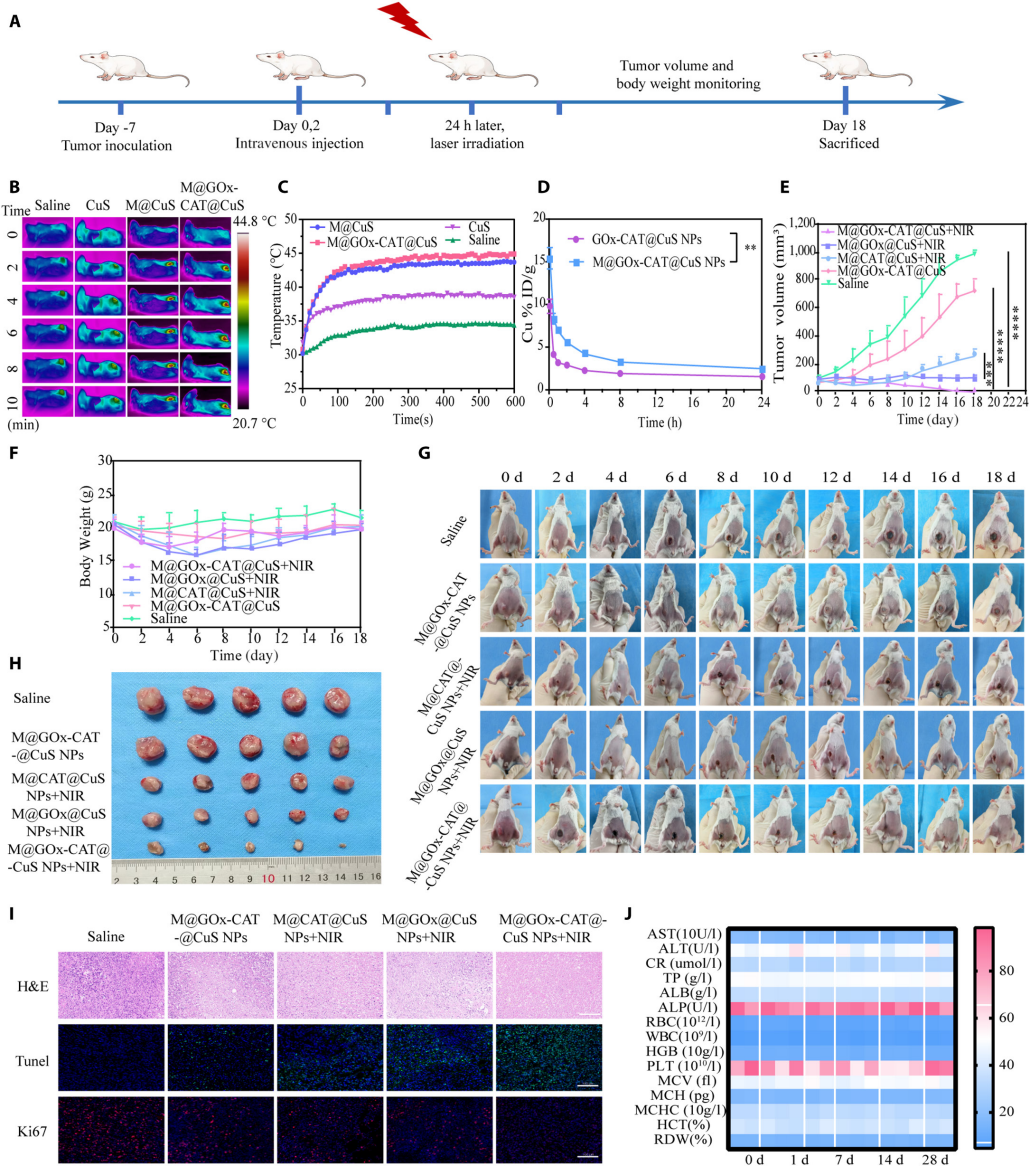
In vivo synergistic antitumor evaluation of M@GOx-CAT@CuS NPs. (A) Schematic illustration of the antitumor treatment process in 4T1 tumor-bearing mice. (B) Infrared thermal images and (C) heating curves of tumors treated with different groups of NPs for 24 h with 808-nm laser irradiation (1.5 W/cm^2^, 10 min). (D) Evaluation of the pharmacokinetics of GOx-CAT@CuS NPs and M@GOx-CAT@CuS NPs by ICP-OES (mean ± SD, *n* = 3). (E) Tumor growth curves and (F) body weights of 4T1 tumor-bearing mice in different treatment groups (mean ± SD, *n* = 5). (G) Images of representative mice from different treatment groups at different times. (H) Representative images of resected tumors collected on 18 d (mean ± SD, *n* = 5). (I) H&E-, TUNEL-, and Ki67-stained images of tumor tissue from different treatment groups. Scale bar: 100 μm. (J) Routine blood analyses and blood biochemistry measurements at different time points after intravenous injection of M@GOx-CAT@CuS NPs (2.5 mg/ml, 200 μl). Significant difference is regarded as ^**^P < 0.01, ^***^P < 0.001,^****^P < 0.0001.

Encouraged by the effects of ex vivo cell therapy, we further set up a 4T1 tumor-bearing mouse model and administered different preparations. As shown schematically in Fig. 8A, the tumor mouse model was set up by injecting 100 μl of cell suspension (1 × 10^6^) into each Balb/c female mouse. After 7 d, the 4T1 tumor-bearing mice were randomized into 5 groups and received saline, M@GOx-CAT@CuS NPs, M@CAT@CuS NPs + NIR, M@GOx@CuS NPs + NIR, and M@GOx-CAT@CuS NPs + NIR by intravenous injection on the 0 and 2 d (2.5 mg/ml, 200 μl), respectively. At 24 h after intravenous injection, 808-nm laser illumination (1.5 W/cm^2^, 10 min) of the tumor region was conducted for mPTT-triggered cascade synergistic treatment. Additionally, infrared thermography was employed to estimate temperature variations in tumors under 808-nm laser exposure (Fig. 8B). According to the monitoring of infrared thermography, the temperature treated by M@GOx-CAT@CuS NPs + NIR could reach 44.9 °C. Compared with M@GOx-CAT@CuS NPs and M@CuS, the warming effect of CuS was comparatively poor, which might be attributed to the fact that CuS lacked the envelope of macrophage membranes and was readily cleared by the reticuloendothelial system, which reduced nanoparticle aggregation within the tumor (Fig. 8C). By monitoring the variations in tumor volume of the mice every 2 d (Fig. 8E, G, and H), we observed that the tumor size of the saline group grew quickly, whereas the tumor growth of the remaining groups was suppressed to a variable extent. Tumors were almost completely eradicated in the M@GOx-CAT@CuS NPs + NIR group, suggesting that mPTT-triggered starvation in combination with CDT can achieve better efficacy than monotherapy.

Besides, we investigated the results of DC cell infiltration within the tumor and in the lymph nodes (Figs. S20 and S21). Remarkably increased DC cell infiltration in the synergistic treatment group of the GOx and CAT cascade triggered immune activation, which effectively inhibited tumor growth. Moreover, the secretion of antitumor cytokines was detected using enzyme-linked immunosorbent assay (Fig. S22). It demonstrated that the M@GOx-CAT@CuS NPs + NIR-treated group had the highest expression of interleukin-6, tumor necrosis factor-α, and interferon-γ, which contributed to the regulation of the immune cells and induced an antitumor inflammatory response. Additionally, mouse body weight remained stable over the treatment period, suggesting relatively low side effects and no systemic toxicity (Fig. 8F).

To estimate the blood circulation of M@GOx-CAT@CuS NPs, copper ion levels in the blood of healthy Balb/c mice were determined by ICP-OES (Fig. 8D). The findings demonstrated that GOx-CAT@CuS NPs were promptly scavenged from the blood, whereas M@GOx-CAT@CuS NPs prolonged the retention time of NPs in the blood due to macrophage membrane coating. Hematoxylin and eosin (H&E) staining indicated that the nucleus of the tumor tissue was notably reduced in the M@GOx-CAT@CuS NPs + NIR group (Fig. 8I), suggesting necrosis of the tumor cells. Ki67 antibody staining to detect the proliferation of tumor cells indicated that the tumor size of the mice in the M@GOx-CAT@CuS NPs + NIR group had the least proliferation of tumor cells (Fig. 8I). Additionally, TdT-mediated dUTP-biotin nick end labeling (TUNEL) apoptosis analysis demonstrated that apoptosis and necrosis were most severe in the M@GOx-CAT@CuS NPs + NIR group (Fig. 8I). For investigation of the biosafety of M@GOx-CAT@CuS NPs (500 μg/ml), it was suspended in erythrocyte solution and no visible hemolysis was observed (Fig. S23). The blood of Balb/c mice intravenously injected with M@GOx-CAT@CuS NPs was subjected to routine blood tests and biochemical indices (Fig. 8J and Fig. S24), and the results showed that the M@GOx-CAT@CuS NPs were biologically safe and nontoxic to mice. In addition, H&E stains of major organs in mice did not show any histological abnormality (Figs. S25 and S26).

## Conclusion

To summarize, we constructed an mPTT-triggered cascade of dual enzymes in a bionic nanoplatform and achieved considerable antitumor effects both in vivo and in vitro. These newly developed macrophage membrane biomimetic nanoparticles were produced by encapsulating GOx-CAT@CuS NPs on macrophage membranes for targeted treatment of breast cancer. Upon uptake by tumor cells, the release of GOx and CAT was triggered by the NIR irradiation, with GOx consuming glucose in the TME and generating H_2_O_2_, and CuS NPs acting as an inducer of the Fenton reaction, catalyzing the decomposition of H_2_O_2_ to generate ·OH. The loading CAT provided oxygen for this cascade reaction, further promoting GOx oxidation and the Fenton reaction, achieving a synergistic combination therapy of mPTT/starvation therapy/CDT. Furthermore, the system was characterized by good biocompatibility, biodegradability, and tumor microenvironment acidic-responsive drug release properties. We believe that this cascade reaction-enhanced biomimetic nanoplatform holds great promise for the effective treatment of tumors.

## Ethical Approval

All animal procedures conducted in the study were approved by Institutional Animal Care and Use of Chongqing Medical University (IACUC-CQMU).

## Data Availability

All data necessary to support these findings are in the manuscript or in Supplementary Materials.

## Supplementary Materials

Supplementary 1 Figs. S1 to S26 References [1–4]
