# Supplementary material for: Metallic Copper-Based Dual-Enzyme Biomimetic Nanoplatform for Mild Photothermal Enhancement of Anticancer Catalytic Activity
Source: Biomater Res. 2024 Jun 5;28:0034. doi: 10.34133/bmr.0034 (PMC11151172; doi:10.34133/bmr.0034)
Supplement: Supplementary 1 — Figs. S1 to S26 References [1–4] [file bmr.0034.f1.docx]

**Supplementary figures**


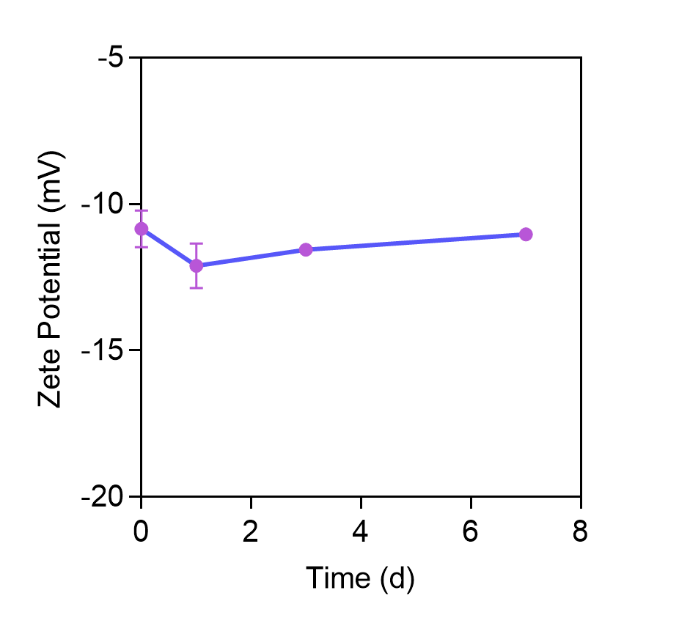

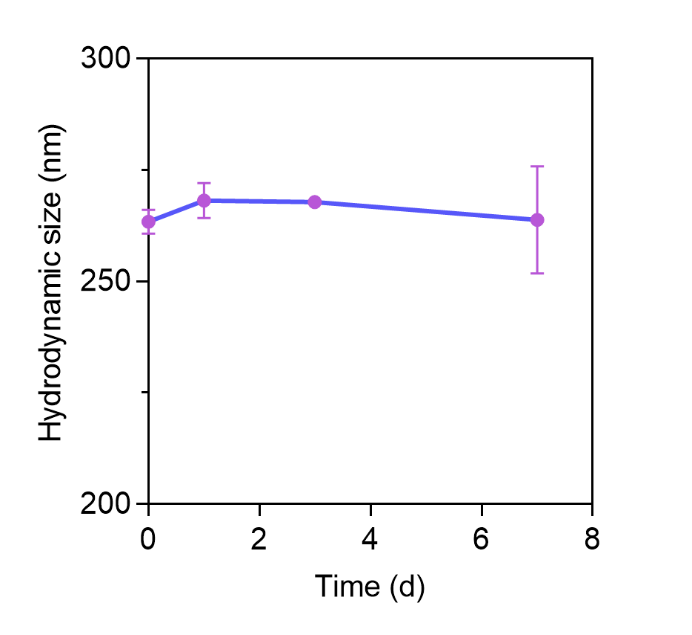


**Figure S1.** Particle size and potential distribution monitoring of M@GOx-CAT@CuS NPs at 0, 1, 3, and 7d (mean ± S.D., *n* = 3).


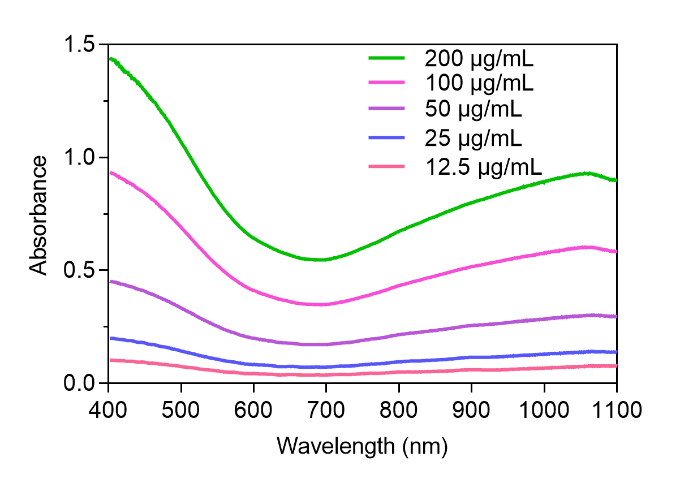


**Figure S2.** UV-vis-NIR absorption spectra of different concentrations of CuS NPs


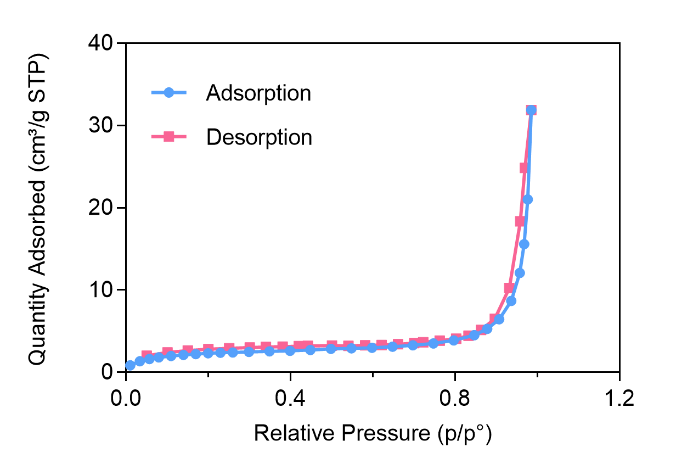


**Figure S3.** N_2_ adsorption-desorption isotherms of CuS NPs.


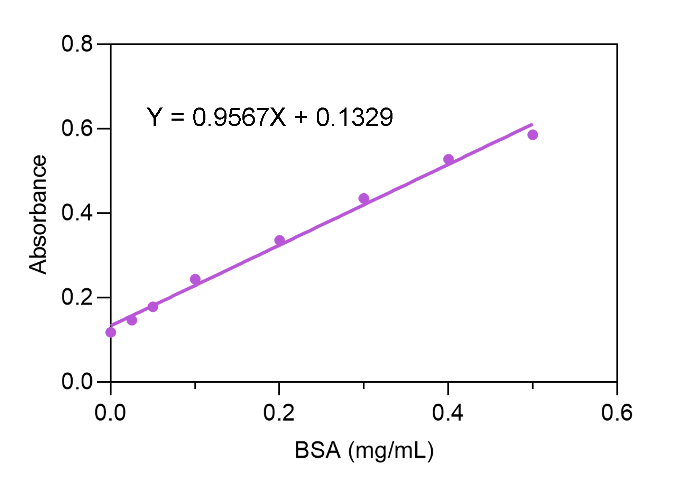


**Figure S4.** Standard curve of BSA at 562 nm (encapsulation rate calculation).

The encapsulation efficiency of GOx and CAT was calculated according to the standard curve in Figure S4:

The absorbance of the supernatant after M@GOx-CAT@CuS NPs washing was 0.1731.According to the standard curve: Y=0.9567X + 0.1329, the concentration in the supernatant was 0.042019 mg/mL. The mass of enzymes in NPs is 4.3697mg. So, the encapsulation efficiency is 4.3697 / 5 = 87.394%.

According to the thermogravimetric analysis (TGA) in Figure 1L, the losing weight

of GOx and CAT in M@GOx-CAT@CuS is 23.15 %.

In the M@GOx-CAT@CuS synthesis process, the dosage of GOx and CAT is 5 mg, and the weight of dried M@GOx-CAT@CuS product is 19.62 mg.

So, the weight of GOx and CAT in M@GOx-CAT@CuS is 19.62 mg × 23.15 % = 4.54 mg. So, according to the TGA, the encapsulation efficiency is 19.62 mg × 23.15 % / 5 (mg) = 90.84%.

The TGA result is in accordance with the Bradford protein assay.


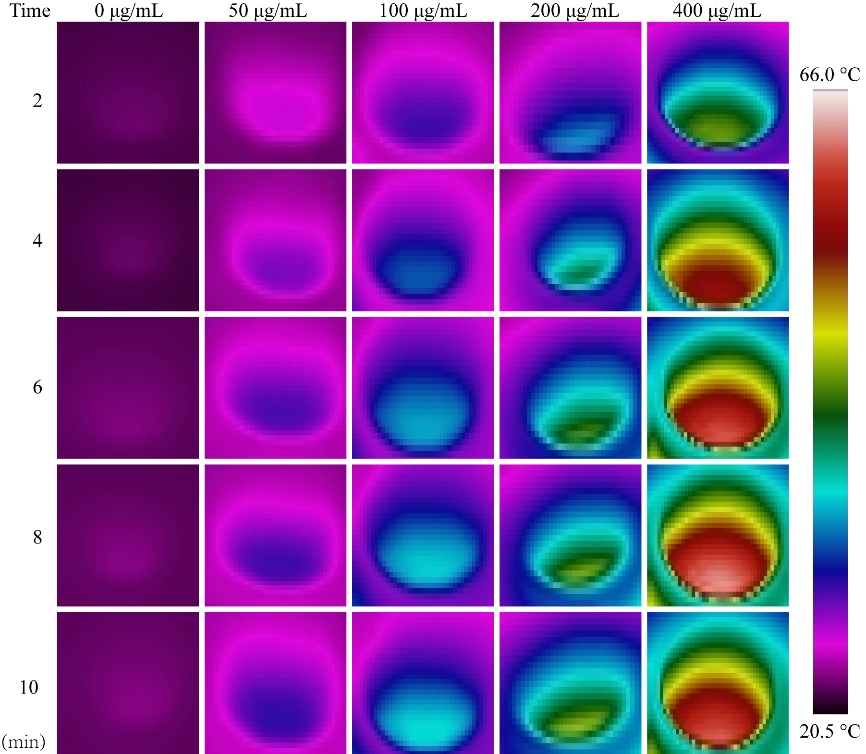


**Figure S5.** Infrared thermograms of different concentrations of M@GOx-CAT@CuS under 808 nm laser irradiation (1W/cm^2^).


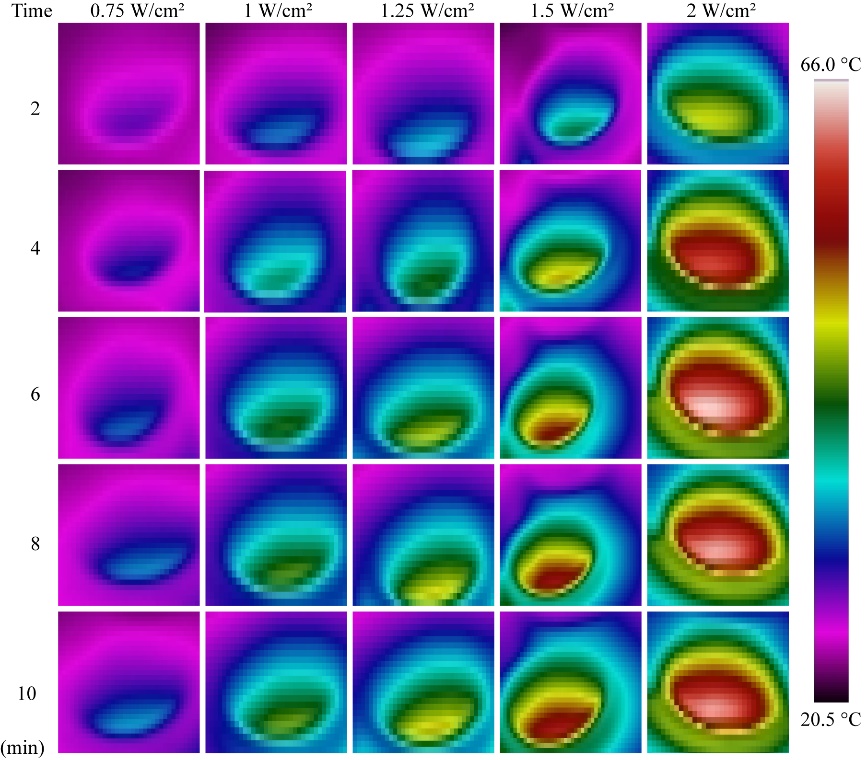


**Figure S6.** Infrared thermograms of M@GOx-CAT@CuS (200 μg/mL) under the irradiation of 808 nm laser of different powers.


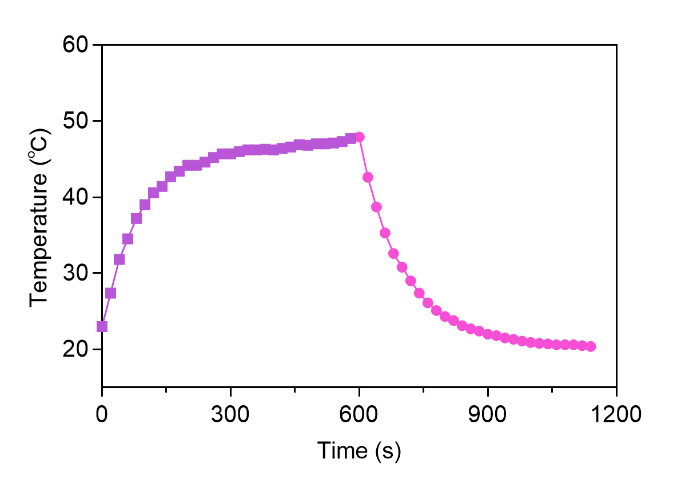


**Figure S7.** Time-temperature curves of heating and cooling of M@GOx-CAT@CuS NPs under 808 nm laser irradiation.


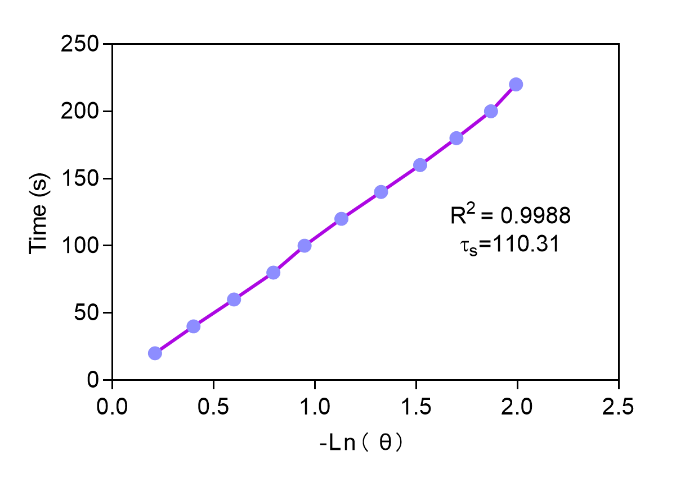


**Figure S8.** The time constant was calculated from the cooling period.


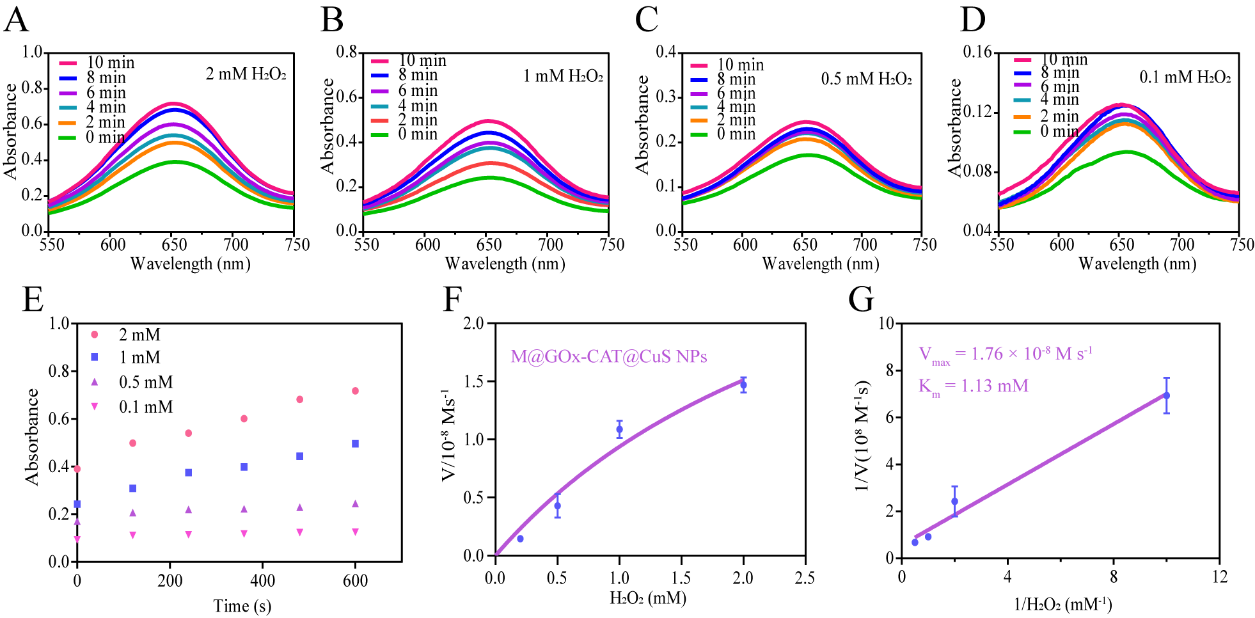


**Figure S9.** Kinetic studies of M@GOx-CAT@CuS with H_2_O_2_ as substrate. (**A-D**) UV-Vis absorption spectra of TMB oxidation catalyzed by the reaction of M@GOx-CAT@CuS NPs (70 μg/mL) with different concentrations of H_2_O_2_ (2 mM, 1 mM, 0.5 mM, 0.1 mM). (**E**) Changes in absorbance of oxTMB at 650 nm with different concentrations of H_2_O_2_. (**F**) Michaelis-Menten kinetic study and (**G**) Lineweaver-Burk plotting of M@GOx-CAT@CuS NPs with hydrogen peroxide as a substrate.


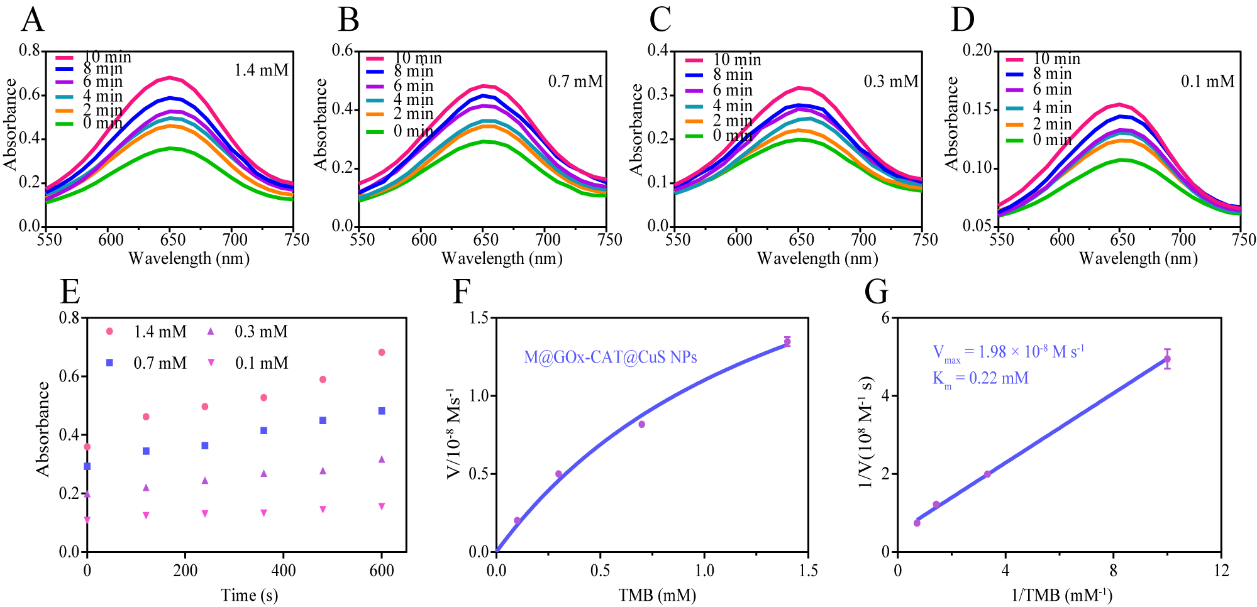


**Figure S10.** Kinetic studies of M@GOx-CAT@CuS with TMB as substrate. (**A-D**) UV-Vis absorption spectra of TMB oxidation catalyzed by the reaction of M@GOx-CAT@CuS NPs (50 μg/mL) with different concentrations of TMB (1.4 mM, 0.7 mM, 0.3 mM, 0.1 mM). (**E**) Changes in absorbance of oxTMB at 650 nm with different concentrations of TMB. (**F**) Michaelis-Menten kinetic study and (**G**) Lineweaver-Burk plotting of M@GOx-CAT@CuS NPs with TMB as a substrate.


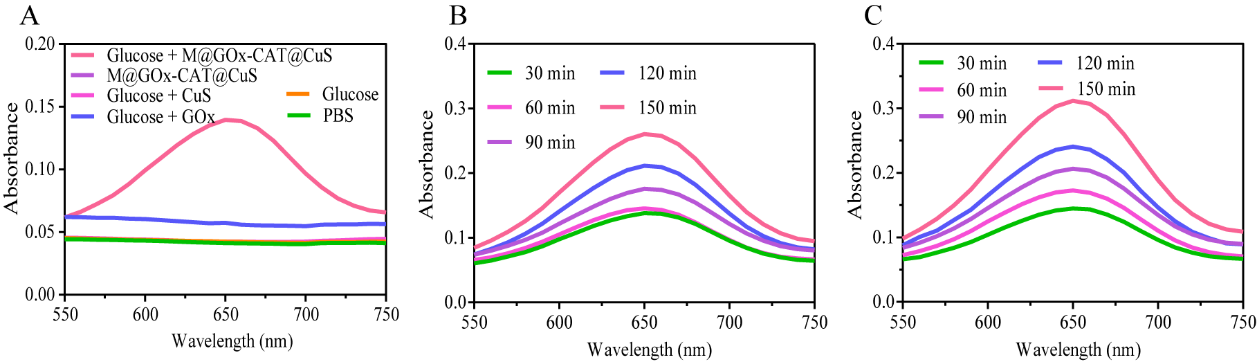


**Figure S11.** Chemical dynamics of M@GOx-CAT@CuS in glucose. **A** UV-Vis spectra of different groups of reaction solutions. UV-vis spectra of M@GOx-CAT@CuS catalyzed TMB in the (**B**) absence or (**C**) presence of laser irradiation


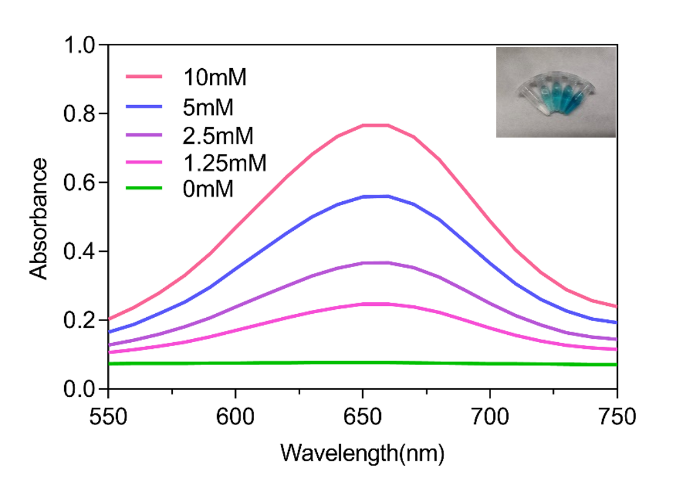


**Figure S12.** UV-Vis absorption spectra of TMB oxidation catalyzed by different concentrations of H_2_O_2_ and M@GOx-CAT@CuS NPs (100 μg/mL, pH=5).


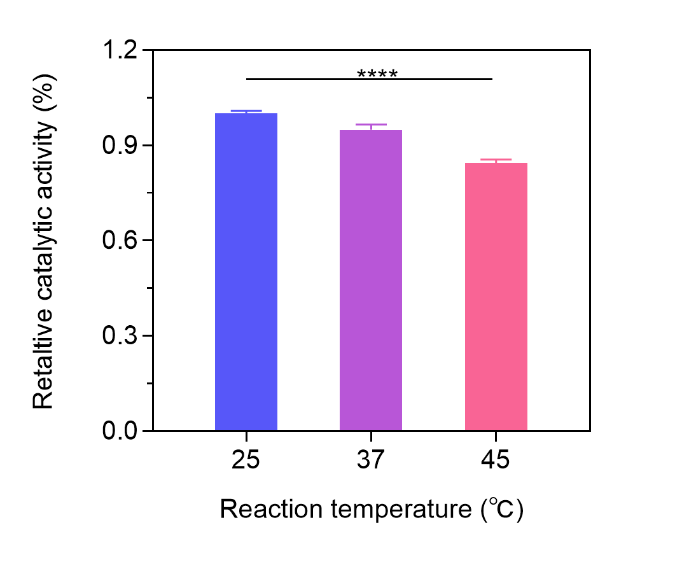

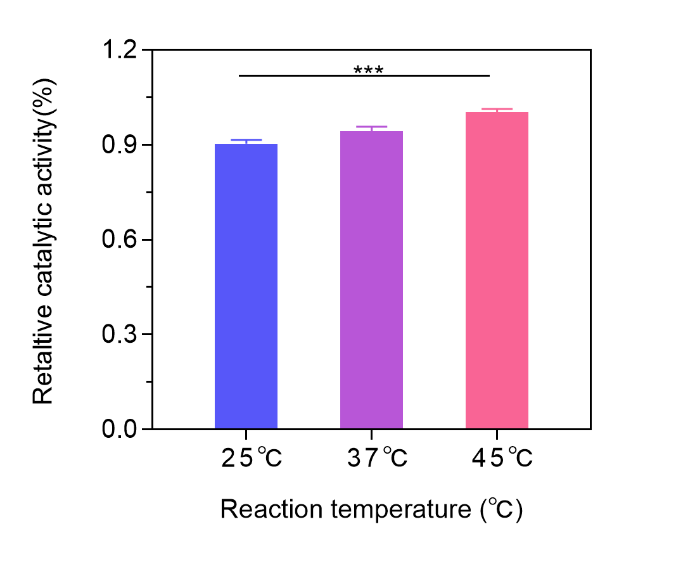


**Figure S13.** Enzymatic activity of GOx (left) and CAT (right) at different temperatures (25°C, 37°C, 45°C) (^***^*P* < 0.001,^****^*P* < 0.0001, *n* = 3).


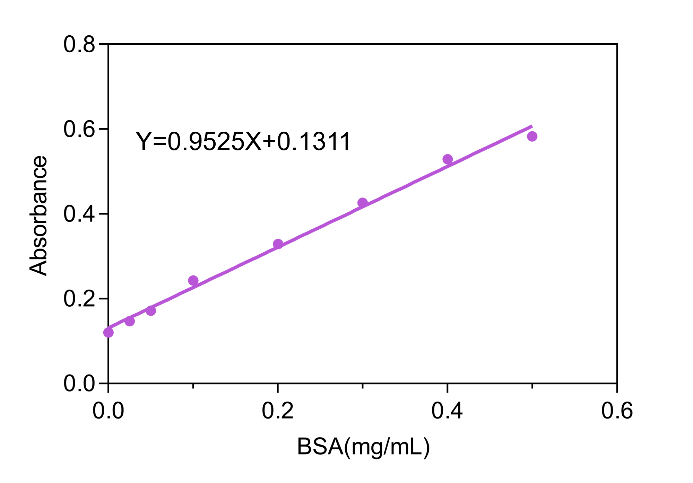


**Figure S14.** Standard curve of BSA at 562 nm (release curve study).

The loading efficiency of GOx and CAT was calculated according to the standard curve in Figure S14:

The absorbance in the supernatant after washing the NPs was 0.272433.

According to the standard curve: Y = 0.9525X + 0.1311, the loading amount of GOx and CAT is calculated as 15.17 mg.

So, the enzyme loading efficiency is 15.17 mg / (20 mg + 15.17 mg) = 43.85%.


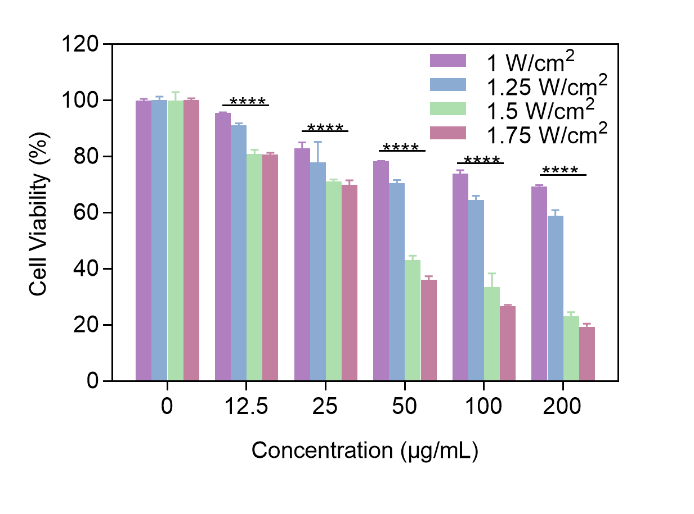


**Figure S15.** Cytotoxicity assessment of CuS NPs at different concentrations under laser irradiation at different power levels (^****^*P* < 0.0001, *n* = 3).


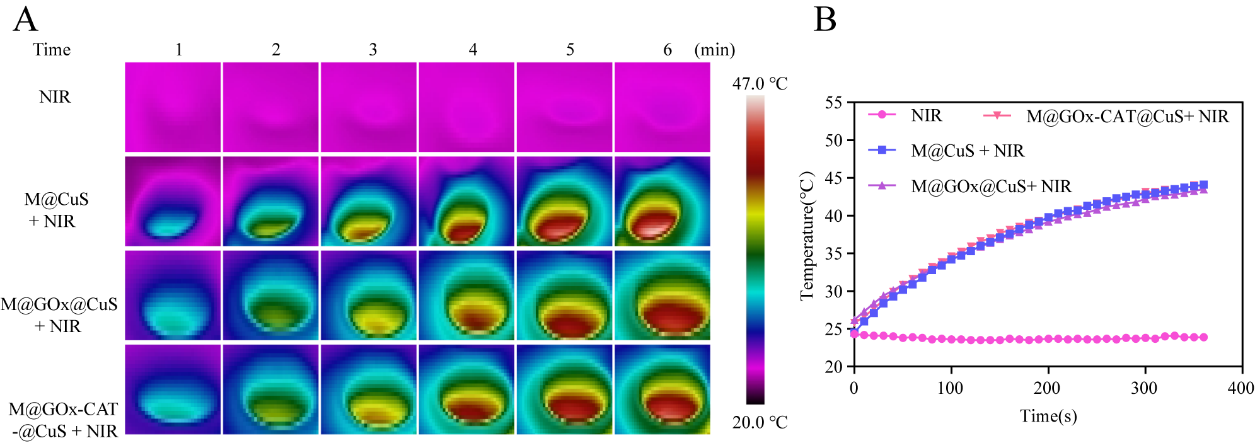


**Figure S16.** Infrared thermograms of cellular combination therapy. **A** Infrared thermal images and **B** heating curves of tumors treated with different groups of NPs for 4 h with 808 nm laser irradiation (1 W/cm^2^, 6 min).


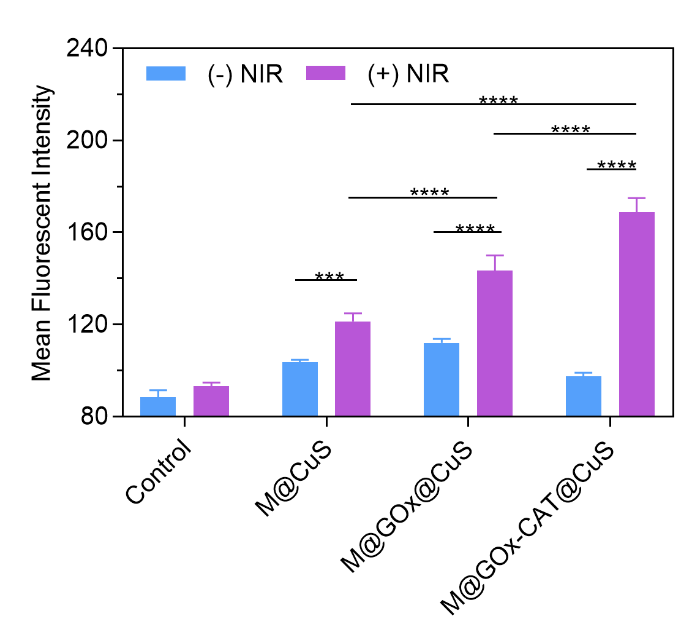


**Figure S17.** ROS fluorescence intensity after treatment of cells in different treatment groups (^***^*P* < 0.001, ^****^*P* < 0.0001, *n* = 3).


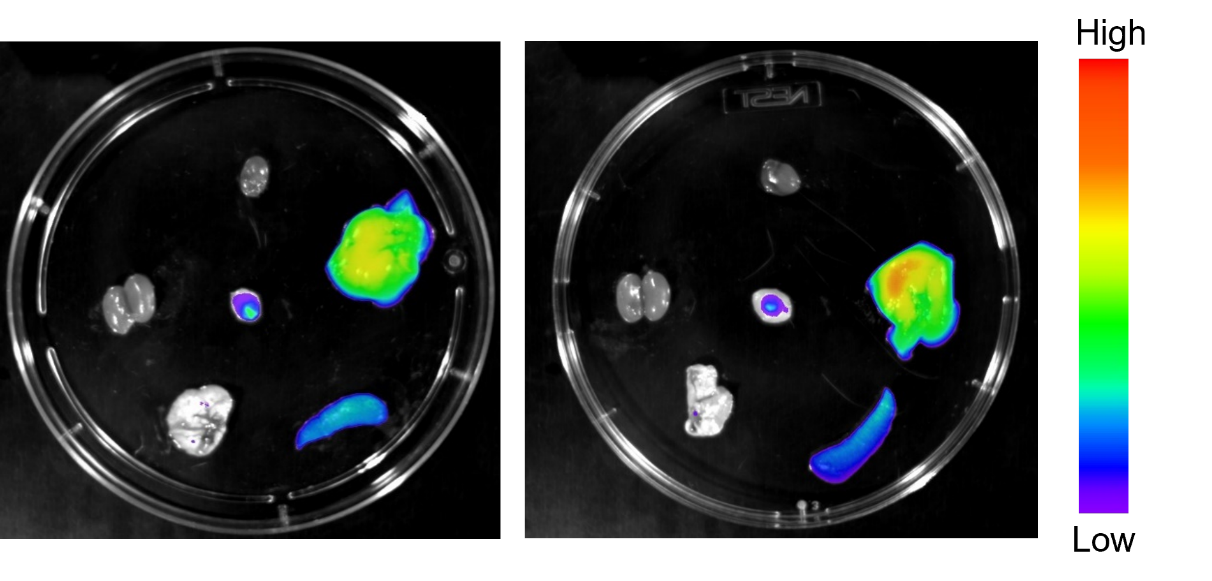


**Figure S18.** Ex vivo fluorescence imaging of mice after 72 h (left: M@GOx-CAT@CuS NPs, right: GOx-CAT@CuS NPs).


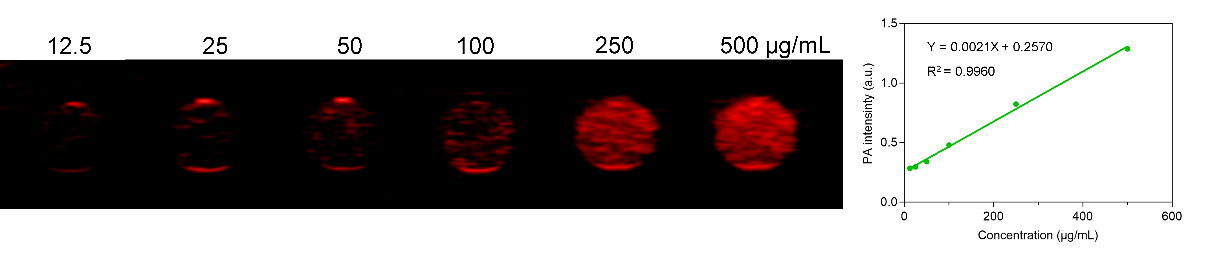


**Figure S19.** In vitro photoacoustic imaging of M@GOx-CAT@CuS NPs at different concentrations and fitting relationships.


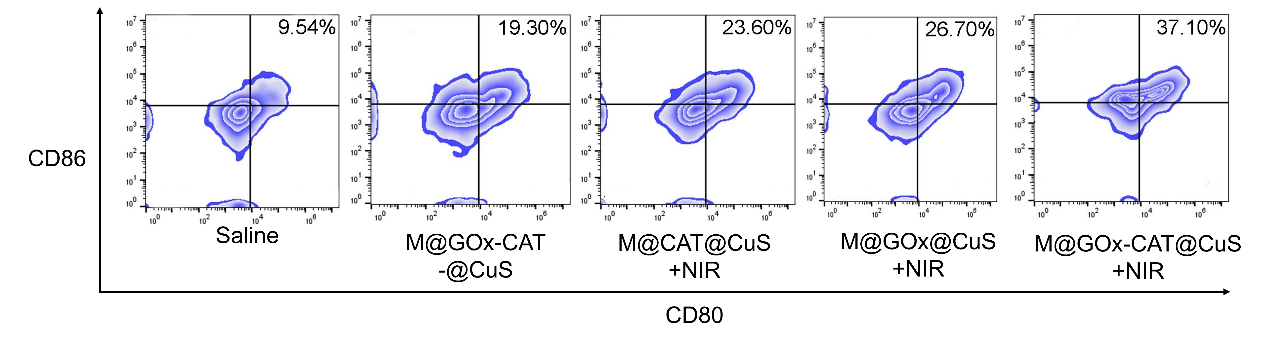


**Figure S20.** Flow analysis of DC cell infiltration results in tumors.


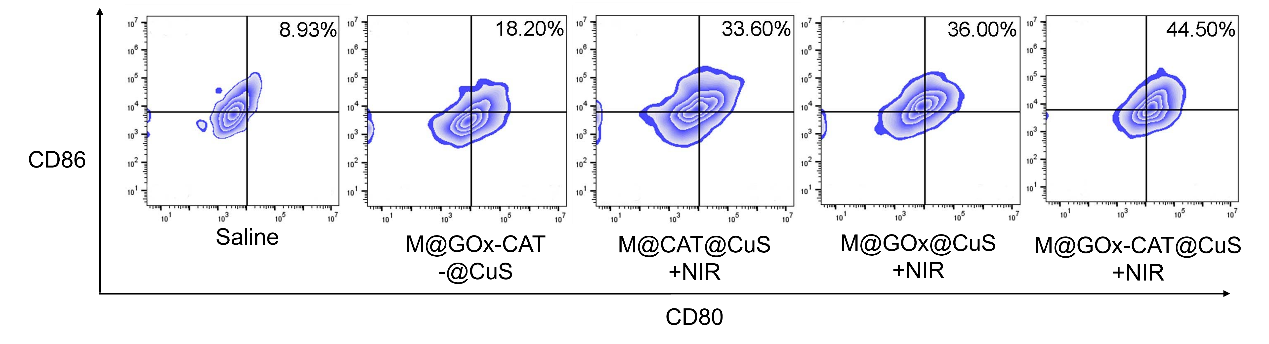


**Figure S21.** Flow analysis of DC cell infiltration results in lymph nodes.


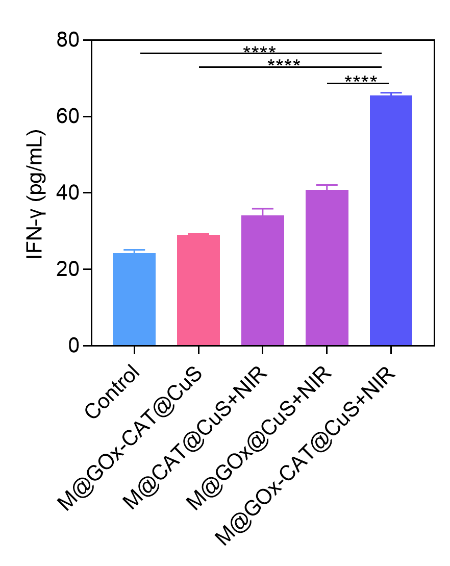

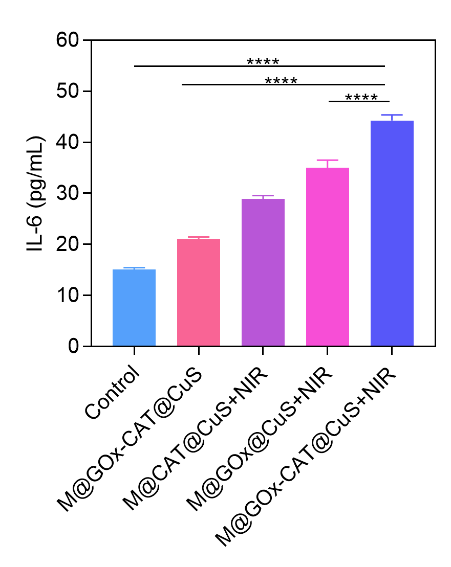

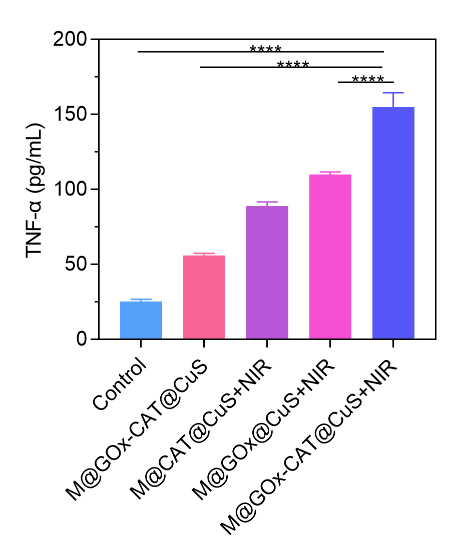


**Figure S22.** IL-6, TNF-α, and IFN-γ levels in serum after different treatments (^****^*P* < 0.0001, *n* = 3)

.


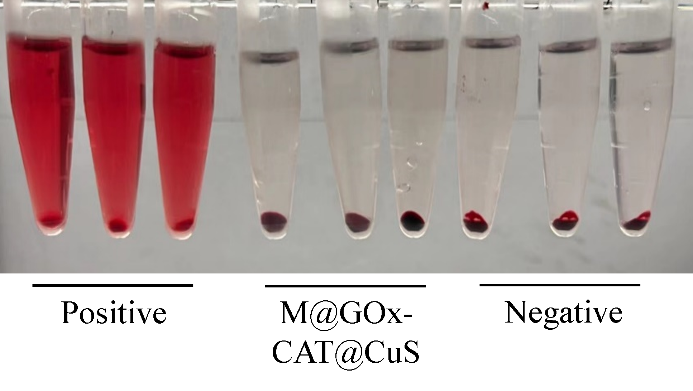


**Figure S23.** Photograph of mouse erythrocytes co-incubated with M@GOx-CAT@CuS NPs (500 μg/mL) for 4 h (groups of three).


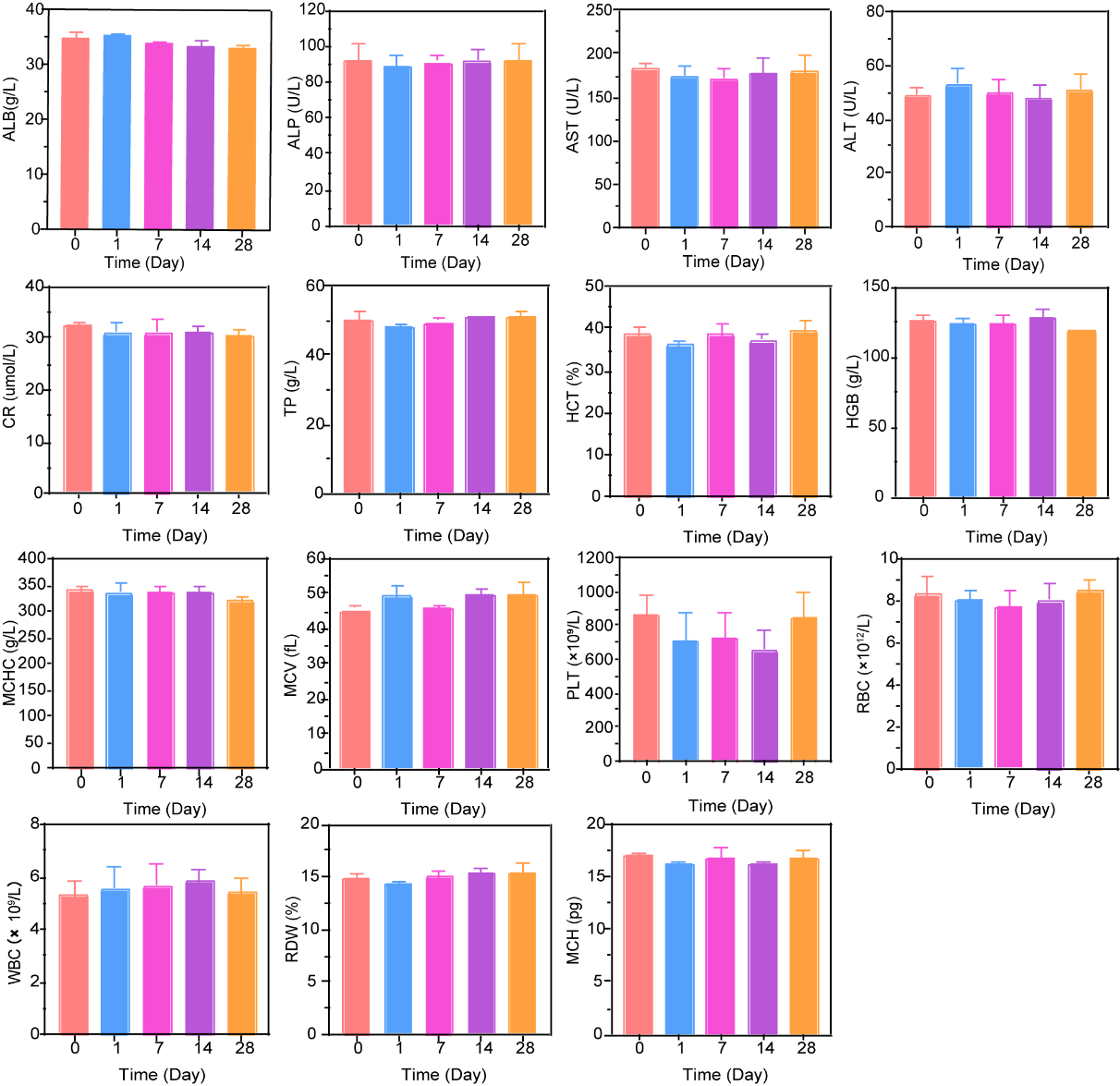


**Figure S24.** Detection of routine blood parameters and biochemical indices in Balb/c mice after intravenous injection of saline (0 d) and M@GOx-CAT@CuS NPs (500 μg) for 1, 7, 14, and 28 d, respectively.


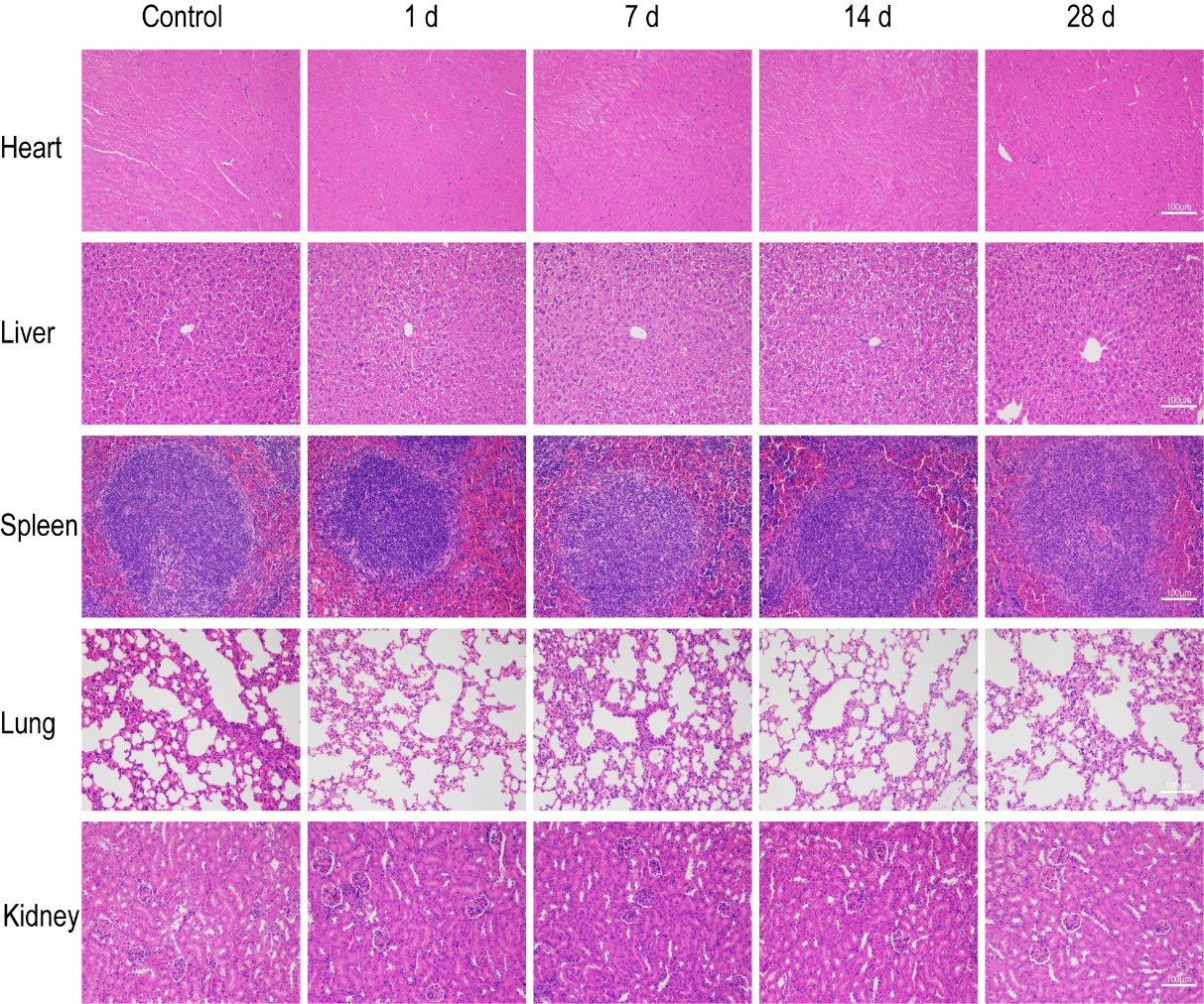


**Figure S25.** H&E staining images of major organs of Balb/c mice after intravenous injection of saline (0 d) and M@GOx-CAT@CuS NPs (500 μg) for 1, 7, 14, and 28 d, respectively. Scale bar: 100 μm.


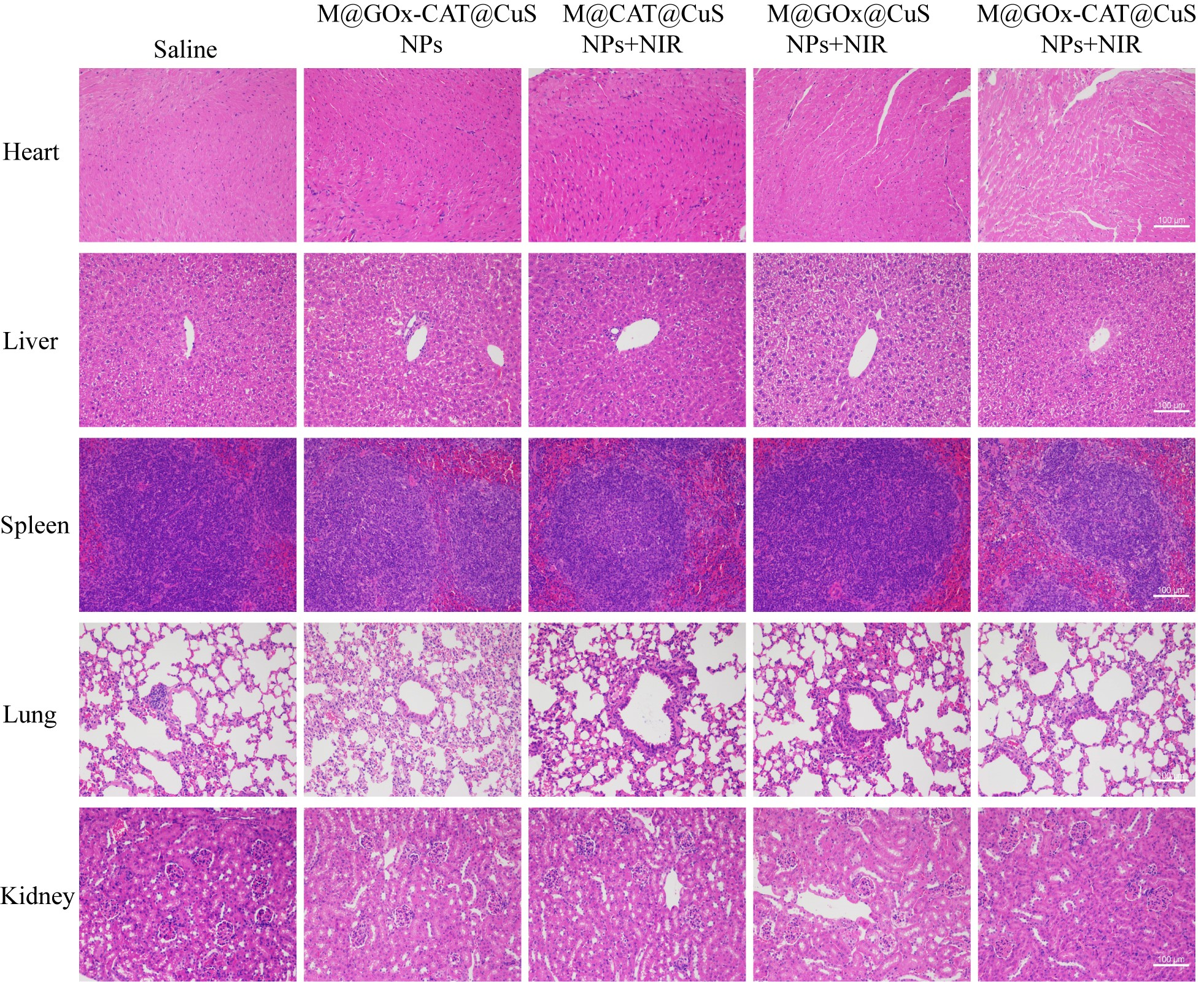


**Figure S26.** H&E staining images of major organs of Balb/c mice after 18 days of treatment in different treatment groups. Scale bar: 100 μm.

**References**

1. Poudel K, Banstola A, Gautam M, Soe Z, Phung CD, Pham LM, Jeong JH, Choi HG, Ku SK, Tran TH, Yong CS. Macrophage-membrane-camouflaged disintegrable and excretable nanoconstruct for deep tumor penetration. ACS Appl Mater Interfaces. 2020;12(51):56767-81.
2. Wang X, Ding C, Zhang Z, Li C, Cao D, Zhao L, Deng G, Luo Y, Yuan C, Lu J, Liu X. Degradable nanocatalyst enables antitumor/antibacterial therapy and promotion of wound healing for diabetes via self-enhanced cascading reaction. Chin Chem Lett. 2023;34(7):107951.
3. Wu F, Huang C, Sun B, Zhu Z, Cheng W, Chen Y, Liao C, Xu R, Maimaititu’ersun ME, Zhou N, Han F. H_2_O_2_ Self-supplementing and GSH-depleting nanoreactors based on MoO_3–x_@Fe_3_O_4_-GOD-PVP for photothermally reinforced nanocatalytic cancer therapy at the second near-infrared biowindow. ACS Sustain Chem Eng. 2022;10(19):6346-57.
4. Zhang J, Sun B, Zhang M, Su Y, Xu W, Sun Y, Jiang H, Zhou N, Shen J, Wu F. Modulating the local coordination environment of cobalt single-atomic nanozymes for enhanced catalytic therapy against bacteria. Acta Biomater. 2023;164:563-76.
